# Syntactic language change in English and German: Metrics, parsers, and convergences

**DOI:** 10.1371/journal.pone.0346096

**Published:** 2026-04-28

**Authors:** Yanran Chen, Wei Zhao, Anne Breitbarth, Manuel Stoeckel, Alexander Mehler, Dominik Schlechtweg, Steffen Eger

**Affiliations:** 1 Natural Language Learning and Generation Lab (NLLG), University of Technology Nuremberg, Nuremberg, Germany; 2 University of Aberdeen, Aberdeen, United Kingdom; 3 Ghent University, Ghent, Belgium; 4 Text Technology Lab (TTLab), Goethe University Frankfurt, Frankfurt, Germany; 5 Institute for Natural Language Processing, University of Stuttgart, Stuttgart, Germany; European Commission, ITALY

## Abstract

Syntactic language change has gained increasing attention in recent years. Previous computational work based on dependency relations has focused on diachronic trends in dependency distance, which measures the linear distance between dependent words, using dependency trees automatically predicted by a dependency parser (mostly the Stanford CoreNLP parser). In this work, we introduce a set of 15 syntax metrics that extend the analysis beyond linear distance by incorporating both linear and tree graph properties of dependency trees, such as tree height and degree. Besides, we propose a multi-parser approach to reduce the impact of using specific parsers, thereby increasing the robustness of the detected language changes. Through a cross-lingual investigation of English and German in parliamentary debates from the last 160 years, using 6 different parsers (CoreNLP and five newer alternatives), we demonstrate that: (1) Relying on one single parser can be problematic, as the agreement on predicted trends can be low across parsers. (2) Our set of metrics can capture subtle patterns of syntactic changes. Our analysis shows that syntactic change over the time period inspected is largely similar between English and German, with only 2.2% of cases yielding opposite trends in these metrics. (3) We also show that changes in syntactic metrics seem to be more frequent at the tails of sentence length distributions and often move in opposite directions for short and long sentences. To our best knowledge, ours is the most comprehensive computational analysis of syntactic language change using modern NLP technology in recent corpora of English and German.

## 1 Introduction

Many studies have shown that human languages are being optimized in the direction of lower complexity and higher efficiency in terms of communication [1–3]. Among others, syntactic dependency distance, i.e., the linear distance between syntactically dependent words, is often interpreted as an indicator of language processing difficulty and working memory load in humans [4–7]. The corresponding optimization principle, is well-established, i.e., dependency distance minimization (DDM), which refers to the tendency/preference of syntactically related words being placed closer to each other.

A large array of works have confirmed the existence of DDM in corpora of natural languages [4,7–9]. On the one hand, studies suggest that the mean dependency distance (or its variants) of a real sentence is shorter than that of random baselines (e.g., sentences with random word order) and this may be a universal property of most natural languages [2,8,10]. On the other hand, there is also evidence that dependency distance decreases diachronically [6,7,11–14].

**Fig 1:**
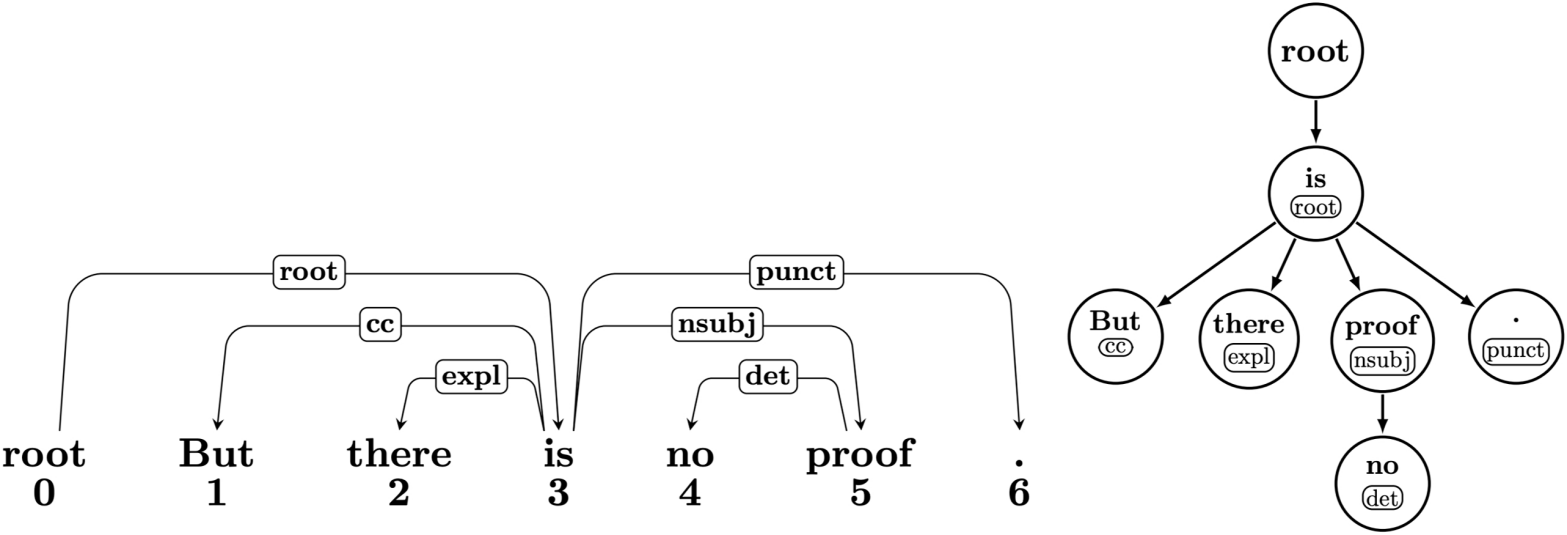
Dependency relations of sentence “But there is no proof”. in linear order (left) and tree graph (right).

Recent diachronic investigations use dependency parsers to automatically parse sentences extracted from corpora covering a large(r) time span, and trace variation in dependency distance over time [e.g., 11, 7, 13]. The main advantage of such studies over approaches that leverage human annotated treebanks [e.g., 15] is that they do not require expensive manual dependency annotations and thus allow for exploring arbitrary, sufficiently large, diachronic corpora. However, to our best knowledge, all of the previous studies rely on a single dependency parser, mostly the Stanford CoreNLP parser [16], which was published nine years ago. Moreover, previous works only focus on linear dependency distance, but as Fig 1 (right) shows, dependency relations can also be structured as a rooted directed tree, allowing for further exploration of syntactic structures based on their natural tree graph properties. Further, the majority of relevant studies are restricted to English, with the exception of [14], who investigates DDM (a.o.) in both English and German (from 1650 to 1900). She compares the trends in scientific and general language, where the texts for general language span multiple genres, such as news and fiction.

In this work, in methodological terms, we go far beyond previous work, introducing a set of 15 syntax metrics that are relevant to DDM and/or based on tree graphs (§5). Besides, instead of relying on one dependency parser, we base our observations on 6 different parsers. In a cross-lingual study of English and German, with a focus on a homogeneous and directly comparable genre of political debates for the last 160 years, we first examine the potential data noise (e.g., OCR errors) in our historical corpora that might impact the parser accuracy and bias the analysis (§3). Then, we inspect the parsers’ legitimacy in our historical use case, as they are mostly only trained and evaluated on modern treebanks, which cover a small recent time period and may exhibit substantially less data noise compared to diachronic corpora (§4). Subsequently, we investigate whether different parsers yield the same trends regarding language change and observe the syntactic changes detected (§6).

Our main research questions are:

- RQ1 (parsers): Are parsers trained on modern treebanks reliable to parse (our) historical data, which is affected by OCR and spelling error changes — especially the German data?
- RQ2 (parsers): When predicting trends of syntactic language change, can one rely on the predictions of a single parser?
- RQ3 (languages): Do English and German mostly change similarly regarding syntax (convergence) or do they have divergent patterns of syntactic language change?
- RQ4 (metrics): How do English and German change when looking at syntactic dependency graph properties beyond mean dependency distance?

Overall, our main contributions are thus methodological ones, reflected in RQ1, RQ2 and RQ4. We make our code and data available at https://github.com/cyr19/syntaxchange.

## 2 Related work

The current paper connects to language change with a focus on syntactic change based on DDM.

### Language change

Language change has been researched for a long time along multiple dimensions (and in multiple communities), including semantic, syntactic, morphological change etc. **Semantic change** investigates the changes in word meaning over time. For example, [17] document semantic change in the words “boy” and “girl”: Girl used to be a term for young people of either sex, while boy described male servants; the present meanings of “girl” and “boy” were first observed in the 15th and 16th centuries, respectively. Earlier works on studying semantic change relied on methods like latent semantic analysis [18] or treated semantic change detection as a diachronic word sense analysis problem [19,20]. More recent studies have leveraged static or contextualized embeddings [21–27], jointly trained across varying periods [21,22,28–30], or independently trained for different periods [31]. The latter scenario then involves remapping embeddings for different periods into one shared vector space [31] or inducing the second-order embeddings, which represents the words’ distance to a reference vocabulary [32,33]. **Morphological change** focuses on how word formation and inflection change over time [34]. For example, it has been shown that in most Germanic languages, including English, case morphology has gradually declined over time [35]. [36] explore case changes in Icelandic, using historical corpora annotated with part-of-speech and other morphological information. Changes in verbal inflection have been explored for many languages. For example, more frequently used English verbs have undergone faster regularization [37]; and verb inflection tends to become weaker in German [38] and Dutch [39] over time (though typically in verbs with lower token frequency; [40,41]). [42] further confirm inflection decay, i.e., languages become less inflectional, in Modern English, using entropy-based algorithms. **Syntactic change** has been discussed for changes in specific syntactic patterns and/or metrics reflecting language complexity. For instance, [3,14,43,44] investigate syntactic shifts in scientific English and/or German, observing several phenomena, such as the tendency towards increasingly heavy/complex noun phrases and a simpler sentence structure with decreasing usage of clauses over time. Similar phenomena were also observed in [45–48]. One of the widely adopted approaches to model language complexity revolves around syntactic dependency distance. Studies have indicated that humans tend to position syntactically related words closer together [13,15,49,50]; this hypothesis/phenomenon is called DDM. Recent work has also proposed operationalizing language (syntactic) complexity using information-theoretic notions of compressibility (description length), i.e., approximations to Kolmogorov complexity [51–54]. Such metrics aim to capture hierarchical/structural properties in addition to linear parsing distances.

### Dependency distance minimization

The most relevant approaches to ours are those based on sentential dependency structures, where (a.o.) the dependency distance and the corresponding principle (i.e., DDM) have been researched extensively. From a **synchronic** perspective, many studies have shown that the mean dependency distance (*mDD*) (or variants) of a real sentence is shorter than that of some random baselines: for example, [55] and [56] observed that the mean dependency distance of Romanian/Czech and Chinese sentences is significantly shorter than that of random sentences, respectively. [5,8] provide large-scale evidence of DDM, which suggests that DDM may be a universal property of human languages. They use treebanks of different languages from various linguistic families to perform the tests and show that the dependency distance of real sentences is shorter than chance. More recently, [2] analyze the optimality (based on dependency distance) of 93 languages from 19 linguistic families. Their results imply that 50% of human languages have been optimized to a level of at least 70%. Compared to English, German has been less optimized according to this study. This finding is in line with [10], who observe that DDM has a much weaker impact on German than on English. Though clearly mystified by this finding (or rather, its diachronic stability), [10] speculate that this may be attributed to the well-known fact that English has strict SVO (subject-verb-object) as basic word order, whereas German is an SOV (subject-object-verb)-language with verb fronting in main clauses, and that therefore, clausal constituents in German vary in their dependency distance to each other in main and embedded clauses.

On the other hand, **diachronic** investigation of dependency distance has received an increasing interest in the past few years [e.g., 6,12,13]. [49], automatically converting the constituency annotations to dependency annotations, finds that the total dependency distance of English sentences decreased from the 9th century to the 15th century (from Old English to Early Modern English). In light of the differences between German and English regarding DDM, the fact that German seems to be less affected by it [10], and the possibility of this having to do with the known typological word order differences, it is probably no coincidence that this decrease in DDM in historical English should have happened exactly at the time when the language became less German-like, losing SOV-order with V-to-I movement (=verb fronting) [57]. [15] compare Latin / ancient Greek from different time periods using manually annotated dependency treebanks, which contain around 1k–10k sentences for each period, finding that languages from the earlier periods exhibit lower DDM levels against the optimum than those from the later periods.

More recent studies leverage dependency parsers to automatically parse sentences extracted from diachronic corpora, and then base their observations on the parsing results [6,7,11–14]. An important benefit of such approaches is that they do not require expensive dependency annotations; therefore, arbitrary corpora beyond treebanks can serve as the research resource. [11,12] investigate the diachronic trend of dependency distance based on the State of the Union Addresses corpus, which contains the political speeches of 43 U.S. presidents from 1790 to 2017. They show that the *mDD* and its normalized variant (*nDD*) [58] of American English sentences are decreasing over time. While [11] observe a consistent downward trend across different sentence length groups, [12] additionally divide the sentences of ≤ 10 words (the shortest sentence group in [11]) into two subgroups: sentences of 0–4 and 5–10 words, finding the existence of an anti-DDM phenomenon for the 0–4 group, i.e., DDM does not apply to short sentences because of the constraints by other language optimization principles [59]. [6,13] compare the diachronic trend of *mDD* and/or *nDD* for the past 100–200 years across varying text genres, based on the COHA corpus, which contains texts in American English across 4 domains: News, Magazine, Fiction, Non-fiction. Their findings are often inconsistent. E.g., [6] find a downtrend of *mDD* only for the Fiction genre but an upward trend for the News and Magazine genres during the period 1900–2009. In contrast, [13] observe a continuous decrease in *mDD* and *nDD* for the Magazine genre spanning from 1815 to 2009. Nevertheless, both of their results imply that the diachronic trends of *mDD* vary for different genres. [7] explore diachronic shifts in syntactic patterns for scientific English, leveraging the Royal Society Corpus [60], which contains scientific publications from 1665 to 1996. They also observe a decrease in *mDD* over time. As follow-up work, [14] comprehensively investigates language optimization for scientific English and German from 1650 to 1900, in comparison to that for general language. She finds that while the *mDD* for scientific language decreased over time, *mDD* for general-domain language does not show a decrease over time during the same period.

Importantly, we need to differentiate between syntactic change at a “deeper” level, e.g., OV to VO as it happened between Old and Middle English [57] from more superficial changes having to do with norms or style, i.e., the choice of different options equally available in a given system. For instance, within English, both active and passive sentences are systematically possible, and it is to some extent a matter of stylistic choice (but also of factors such as information structure) which one is used. The importance of considering genre differences has been pointed out in previous literature [50]. Such differences are synchronically also situated at the level of stylistic choices. For instance, the more condensed structures in scientific English that [7] found are such stylistic choices: more complex noun phrases instead of more complexity at the clausal level. Such stylistic choices also concern the distinction between conceptually more ‘written’ and more ‘oral’ styles, colloquial vs. formal etc. – again choices within one language system [61–63]. Parliamentary debates are interesting in this respect because they consist of often prepared written statements that are delivered orally, i.e., they combine conceptual written style with medial orality. In that delivery, there may be diachronic changes in stylistic preferences, such that a too strongly conceptually written style comes to be less preferred over time. Existing literature on the colloquialization of parliamentary debates [64–67], while suggesting that this is indeed a plausible hypothesis, is mostly restricted to different varieties of English, and focuses mostly on changes at a more lexical level [67,68]. None of them leverages the properties of syntactic dependency parsing to trace syntactic trends. Some stylistic changes in political debates have also been attested across languages such as decreases in formal language and increases in confident language [69]. Of course, stylistic choices, though perhaps more “superficial” than system-level syntactic change, still constitute syntactic variation, and identifying metrics able to pick up on them is one of the aims of the current paper.

Existing studies have three main limitations: (1) all of them rely on one single parser, mostly the Stanford CoreNLP parser [16] with an exception that [14] leverages the UDPipe parser [70]. [71] have shown that *different annotation schemes* may have an impact on dependency distance; in our work, we explore whether *different parsers* yield the same outcomes regarding our diachronic investigations. (2) Some of the works may report biased results due to improper sentence grouping. We show a clear trend of shorter sentences appearing more frequently in later periods, consequently, the average sentence length per decade is decreasing over time. [13] do not differentiate sentences by lengths and report the diachronic trends on all sentences. Lei and Wen [12], Liu et al. [11] divide the sentences into groups having 1–10 (0–4, 5–10), 11–20, 21–30, and 31 + words, and analyze trends for each group separately. However, we demonstrate that even when restricting the maximum difference in sentence lengths to 3 tokens within a group, a slight downtrend in sentence length over time persists (refer to §7.1). Given the substantial length disparity within a group in their works (e.g., 10 for group 11–20), and lacking information on the length distribution in their data, it is hard to tell whether the observed downtrend of *mDD* is the effect of decreasing sentence length or really due to DDM. Those facts may cast doubts on the legitimacy of their findings. (3) Almost all of the works focus on English change, except for [14], who compares the changes in scientific and general English and German. The dataset for general languages used in [14] contain texts in multiple genres like fiction and news. Nevertheless, as [6,13] suggest, trends of *mDD* may differ for various text genres. Instead, in this work, we mainly focus on one specific genre, namely *political debates*, across both languages; in §7, we also briefly inspect the generalizability of our approach to other domains.

## 3 Data preparation

### Corpora

In this work, we use corpora consisting of political debates and speeches in German and English to keep the factors ‘genre’ and ‘time’ constant, and thus ensure comparability between the two corpora. For **English**, we select the **Hansard** corpus, which comprises the official reports of UK parliament debates since 1803. To compile the data for the time period 1803–2004, we extract XML files from the Hansard archive, retaining every section labeled as “debate”. Data from 2005 to April 2021 is obtained from a Zenodo repository. For **German**, we utilize the **DeuParl** corpus published by [72]; it contains plenary protocols from both the Reichstag (the former German parliament, until 1945) and the Bundestag (the current German parliament), covering the period from 1867 to 2022.

### Preprocessing

**Fig 2:**
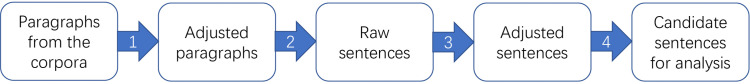
4-step pipeline for sentence extraction from the corpora: 1. *paragraph-level preprocessing*, 2. *sentence segmentation with Spacy*, 3. *postprocessing*, 4. *filtering.*

To extract sentences from the corpora, we employ a 4-step preprocessing approach, outlined in Fig. 2. Initially, we conduct preprocessing at the paragraph level, with the aim of providing cleaner inputs for the sentence tokenizer. For example, as Table 1 (first column) shows, in the original data of Hansard, the page numbers are often not excluded from the sentence due to OCR errors, which occupy an individual line (“[238”). In DeuParl, the text is often split into lines by periods, no matter whether they serve as the ending punctuation. As shown in Table 1, “z.B.” (English: “e.g”) is accidentally split into two lines (third column); a similar case exists for “27. März” (English: “27. March”) in the last column. With paragraph-level preprocessing, we delete the line break and the page numbers (for example) before applying the sentence segmentation. In the case of DeuParl, which is in plain text format, we first divide each file into paragraphs of 50 lines, in order to ensure not to exceed the maximum input length of the sentence tokenizer. For Hansard, the paragraphs are naturally organized within the XML files. Subsequently, we utilize Spacy [73] to segment the paragraphs into sentences. The next step involves postprocessing on the segmented paragraphs, primarily focused on correcting errors stemming from the sentence tokenizer. Furthermore, we find inconsistent usage of semicolons as sentence-ending punctuation. For example, in the UD treebanks, semicolons are occasionally permitted as the concluding punctuation of sentences; at other times, multiple individual sentences connected by semicolons are treated as one sentence. However, the sentence tokenizer tends to split the sentences by semicolons. With simple postprocessing, we manage to correct such segmentation errors by concatenating sentences separated by semicolons. Finally, we apply a filtering process to exclude texts that may not qualify as complete and standalone sentences; this is achieved through the following simple and intuitive rules:

**Table 1:** Examples of problematic data. Line breaks in the table signify actual breaks in the data, with problematic texts highlighted in red.

Hansard		DeuParl	
“The Earl of Liverpool [...] [238 stance, when he [...]	Blankets 18,800 13,500 6,360	Ich will Ihnen z. B. sagen, ist [...]	(Bravo!) Rechts.) Reichstag des [...] 20. Sitzung am 27. März 1867.

- Sentences must start with a capitalized character.
- Sentences must end with a period, or a question mark, or an exclamation mark.
- Sentences must contain a verb based on the part-of-speech tags.
- The number of (double) quotation marks must be even.
- The number of left brackets must be equal to that of right brackets.

It is possible that such preprocessing/filtering biases the results later discussed to some extent, as some sentences are ignored through it. however, directly applying sentence segmentation to the original data leads to incomplete sentence segments or data from other parts, e.g., a budget table, as Table 1 (second column) shows, which can result in larger biases for the mismatch with the definition of the dependency parsing task, i.e., parsing the syntactic structure of a sentence, and consequently affect the metrics calculated on a sentence level.

### 3.1 Validation and correction

To validate the feasibility of our preprocessing pipeline and study the influence of data noise on metrics such as dependency distance (discussed in §4.1.4), for each corpus, we apply the pipeline to five randomly sampled paragraphs from each decade, resulting in approximately 150–800 sentences per decade. We then manually review ten randomly selected outputs for each decade and make corrections if any issues are identified. Additionally, the data from the 2020s is merged into the 2010s, given that only partial data is available for the former.

### Annotation

We first check the **German** sentences from DeuParl. Two annotators with an NLP background, one male faculty member who is a German native, and one female PhD student who speaks German fluently, check the outputs. In this phase, we do not have annotation guidelines; the annotators are asked to freely make comments on each text following their intuition and make corrections if they identify any issues in the text; the annotation is done via Google spreadsheet. Subsequently, by inspecting the comments and comparing them to the original PDF files, we standardize and format the annotation scheme. As Table 2 shows, the formatted annotation has 5 columns:

- **(1) is_sent**: A **binary** annotation (‘TRUE’/‘FALSE’) for sentence identification, i.e., whether the text is a sentence (minor issues in the sentence, such as space or spelling errors, are allowed).
- **(2) has_issues**: A **binary** annotation (‘TRUE’/‘FALSE’) about the existence of any issues (only if (1) is ‘TRUE’).
- **(3) issues**: A sequence of labels for the **category of the issues** identified, e.g., spelling errors (only if (1) and (2) are ‘TRUE’).
- **(4) origin_of_issues**: A sequence of labels for the **origin of the issues**. For example, if a spelling error is a consequence of OCR errors, then the “origin” of it should be “OCR”. The labels here are aligned in a one-to-one manner with those in (3) (only if (1) and (2) are ‘TRUE’).
- **(5) correction**: The **corrected sentence** (only if (1) and (2) are ‘TRUE’).

**Table 2:** Illustration of the formatted annotation.

Text	Date	Is sent	Has issues	Issues	Origin of issues	Correction
Ich bitte, daß diejenigen Herren, welche für den Fall der Annahme des Z 33b in demselben die Worte,oder an Druck m anderen öffentlichen Orten” aufrecht erhalten wollen, sich von ihren Plätzen erheben.	1883-4-6	TRUE	TRUE	extra material; symbol; spelling	ocr; ocr; historic	Ich bitte, dass diejenigen Herren, welche für den Fall der Annahme des § 33b in demselben die Worte,oder an anderen öffentlichen Orten” aufrecht erhalten wollen, sich von ihren Plätzen erheben.

We classify the **issues** into 5 categories: *Spelling*, *Space*, *Missing Material*, *Extra Material* and *Punctuation & Symbol*, as shown in Table 3. Additionally, we split the **origins of issues** into 4 categories: (1) *Historic*, which indicates the issues are due to the differences in historical and modern language. For example, ‘daß’ (modern spelling: ‘dass’) is a spelling issue with the *historic* origin. (2) *OCR*, which indicates the issue is an OCR error. For example, the spelling issue in Table 3 results from an OCR error (‘geschloffen’ vs. ‘geschlossen’). Here, the archaic long S in word medial or initial position is misanalysed as ⟨f⟩). (3) *Genre*, which represents issues that exist due to the characteristics of the text genre. In the text of political debates, the language changes between written and spoken language frequently, and also, there exist interjections within a sentence, as shown in the fourth row of Table 3 (Extra Material). (4) *Preprocessing*, which denotes that the issues stem from our preprocessing pipeline, e.g., extra space. For details regarding those categories, we refer to Fig. 17 in S1 Appendix, which demonstrates our annotation guidelines following the refined annotation scheme.

**Table 3:** Categories of issues; each is shown with an example and the corresponding correction. The parts having a certain issue or missing in the sentences are in red.

Category	Example	Correction
Spelling	Das ist die Mehrheit; die Diskussion ist geschloffen.	[...] geschlossen.
Space	Der EVG-Vertrag spreche von der,westlichen Verteidigung,” und im Protokoll der NATO-Staaten sei vom Zusammenschluß der w e s t europäischen Länder die Rede.	[...] westeuropäischen [...]
Missing Material	Ich bin der Meinung — und viele an uns herangekommene Klagen lassen auch darauf schließen —, dass die Auffassung des Reichssparkommissars, dass das Personal der Deutschen Reichspost voll ausgelastet, aber nicht überlastet sei, nicht richtig ist.	add “—”
Extra Material	[...] daß durch den Bund zweierlei Recht für die Norddeutschen geschaffen werden soll, (Sehr richtig!) daß gewissermaßen zweierlei Klassen von Norddeutschen geschaffen werden sollen, (Sehr gut!) eine Selekta, die vermöge ihrer Gesittung [...]	delete “(Sehr gut!)” and “(Sehr richtig!)”
Punctuation & Symbol	Ich bitte, daß diejenigen Herren, welche für den Fall der Annahme des Z 33b in demselben die Worte,oder an Druck m anderen öffentlichen Orten” aufrecht erhalten wollen, sich von ihren Plätzen erheben.	[...] § 33b [...]

Next, two annotators annotate the **English** text from Hansard, following the annotation scheme defined above. As the original PDF files of Hansard are inaccessible, we cannot identify the origins of the issues; thus, this annotation is omitted for Hansard. Among the annotators, one is a male faculty member, while the other is a female Master’s student, both of whom speak English fluently and work in the NLP field.

To check the **agreement** among annotators, 40 German sentences are jointly annotated, 20 for the sentence identification task and 20 for the issue identification/correction task. For English, 30 sentences are commonly annotated for all annotation subtasks. For each binary annotation task, we calculate Cohen’s Kappa [74] for inter-agreement, whereas for the correction task, we compute the edit distance [75] between sentences. Let *s* be the original sentence, and $s_{a}^{\prime }$ and $s_{b}^{\prime }$ be the sentences corrected by annotators A and B respectively. We calculate the edit distance (i) between *s* and $s_{a}^{\prime }$ ($ED(s,s_{a}^{\prime })$), (ii) between *s* and $s_{b}^{\prime }$ ($ED(s,s_{b}^{\prime })$), and (iii) between $s_{a}^{\prime }$ and $s_{b}^{\prime }$ ($ED(s_{a}^{\prime },s_{b}^{\prime })$). If the corrections are similar to each other, (i) and (ii) should be close while (iii) should be small. Overall, the annotators obtained a decent level of agreement, with ∼ 0.7 on binary tasks and the edit distances of (i) = 3.88, (ii) = 3.96, and (iii) = 0.54 for the correction.

### Results

**Fig 3:**
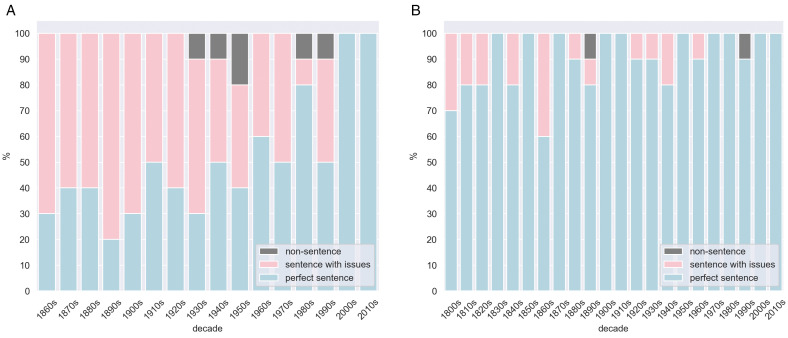
(a) Deuparl (b) Hansard Distribution of the texts identified as perfect sentences (without any issues), sentences with issues, and non-sentences over time.

We show the distribution of the texts identified as perfect sentences (without any issues), sentences with issues, and non-sentences in Fig 3. We observe that: (1) German data has more issues compared to English. For the older time periods, e.g., the first 3 decades in each corpus, 60–70% German sentences are found to contain issues, while there are only 20–30% English sentences found to be problematic. (2) Only a small portion of sentences were identified as non-sentences. Specifically, 6 out of 160 texts (∼3.75%) from DeuParl and 2 out of 220 texts (∼0.1%) from Hansard are found not to be sentences. (3) The proportion of problematic sentences is decreasing over time; for both languages, all sentences after 2000 are flawless.

**Fig 4:**
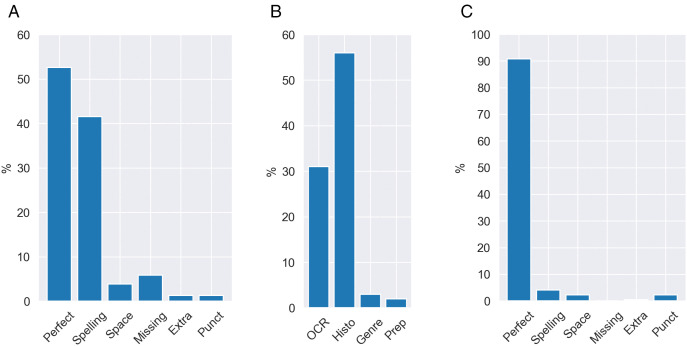
(a) Deuparl – issues (b) Deuparl – origins (c) Hansard – issues (a)/(c): Percentage of perfect sentences and sentences with a specific issue to all texts identified as sentences. (b): Percentage of sentences with issues from a specific origin to all sentences containing issues.

Next, we present the percentages of the sentences having a specific issue in Fig 4a and 4c. Note that one sentence can have multiple issues, so the sum of the percentages does not need to equal 100%. In English data, 90% of the sentences are perfect — only 1%−2% of the sentences have *Spelling*, *Space*, or *Punct* issues. In contrast, only half of the German sentences are issue-free, with over 40% of the sentences having *Spelling* issues and 1%−5% of the sentences containing other issues. Furthermore, we find that *Historic* origin issues dominate in the problematic German sentences—over 55% of those contain at least one *Historic* origin issue, followed by *OCR* origin—∼30% of the flawed sentences have issues raised from OCR errors.

In summary, our annotations suggest that English data from Hansard is substantially less problematic than the German data from Deuparl. Notably, the most prevalent issues in the German data, namely historical spelling and OCR spelling errors, rarely appear in the English data. This leads us to question whether the Hansard Corpus has already undergone preprocessing like spelling normalization. Through contact with the UK Parliament enquiry services, we received feedback indicating that the data has not been preprocessed regarding spelling normalization. As for OCR errors, a statement on their website explicitly mentions that OCR errors are curated once identified. As a consequence, we conclude that English has undergone smaller changes in terms of spelling in the past 200 years compared to German, making German the more interesting (and much less researched) language from a historical perspective.

### 3.2 Dataset construction

**Fig 5:**
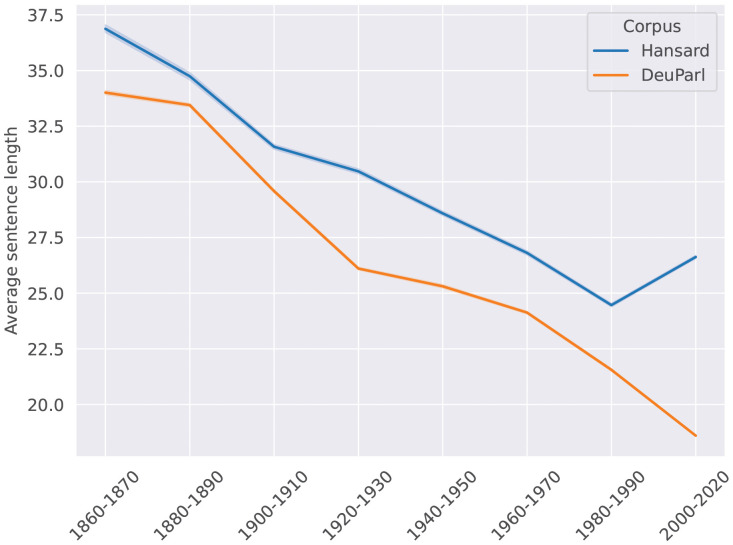
Average sentence length per decade group over time.

We apply the preprocessing pipeline introduced above to get up to 200k sentences for each decade and language. To make the English and German data comparable, we limit the time period for English to 1860–2020. Next, we tokenize the sentences and do part-of-speech tagging using Stanza [76]. The length of sentences is then the number of tokens after tokenization. Fig 18 (in S1 Appendix) and Fig 5 show the distribution of sentence lengths and the average sentence length over time. We see that shorter sentences appear more often in the later periods and thus the average sentence length is overall decreasing over time for both languages. For instance, the average sentence length in DeuParl decreases from ∼35 to ∼20 in the time window 1860–1870–2000–2020. The only exception is the last subtrend for Hansard, where the average length increases by ∼2 tokens. Metrics like *mDD* are highly sensitive to sentence length [58], thus, it is important to control for sentence length when constructing the dataset for observation.

#### 3.2.1 Final dataset.

We select sentences with varying number of tokens for observation: 5, 10, 15, 20, 30, 40, 50, 60, and 70, covering short, middle and long sentences. We aim to observe syntactic changes in sentences of the same length (i.e., same amount of tokens). Nevertheless, to obtain sufficient sentences for each length and time period, we set an offset of 2 tokens for sentence grouping, e.g., sentences of a length from 10 to 12 are grouped together; in addition, we consider decade groups with each spanning 2 decades (except for the last one, which spans 3 decades). For both English and German, the final dataset contains 450 sentences per length and decade group, totaling 32,400 sentences for each language.

## 4 Dependency parsers

Unlike previous approaches, which solely relied on a single parser, typically the Stanford CoreNLP Parser [16], we will base our observations on various parsers with the two most popular design choices: transition-based and graph-based. Transition-based parsers sequentially predict the arc step by step based on the current state, while graph-based parsers globally optimize the dependency tree, aiming to find the highest-scored one. The parsers used in this work include: (1) **transition-based**: CoreNLP [16], StackPointer [77]; (2) **graph-based**: Biaffine [78], Stanza [76], TowerParse [79] and CRF2O [9]. We use the implementation of Biaffine and CRF2O from https://github.com/yzhangcs/parser, StackPointer from https://github.com/XuezheMax/NeuroNLP2, and TowerParse from https://github.com/codogogo/towerparse. We use Stanza of version 1.5.1 and CoreNLP via the client API bundled in Stanza.

### Training sets

We train Biaffine, StackPointer, and CRF2O on the concatenated training splits of the treebanks in UD v2.12 [80] to expose the parsers to more diverse data in terms of text domains, facilitating (potentially) better out-of-domain performance. Among the UD treebanks, only GUM [81] and ParTUT include sentences, some of which are from domains similar to ours, such as political speeches and European Parliament proceedings. We train model checkpoints separately for each language; the number of sentences used for training is shown in the ‘Train’ column of Table 4. The authors of TowerParse released the checkpoints trained on individual treebanks in UD2.5; as they did not provide the training script, we directly take the models trained on the largest treebank for each language, i.e., EWT for English and HDT for German. For CoreNLP and Stanza, we use them with the out-of-the-box settings. Note that the default models in Stanza 1.5.1 were also trained using UD2.12, however, the default German model is only trained on the GSD treebank, and the default English model is trained on the combination of 5 out of 7 training sets in the treebanks.

**Table 4:** Number of sentences in the treebanks used. The data size of the test UD treebank is the sum of all treebanks. The value for the OCR spelling attack is the total across the six attack levels (2k per attack level).

	UD		Target	Adversarial	
	Train	Test		Historical	OCR
en	32,865	7,795	111	–	–
de	166,849	22,356	163	1,948	12,000

### Pre-trained language models

Among the parser checkpoints we trained, Biaffine uses (XLM-)RoBERTa [82,83] models as the encoder, while StackPointer and CRF2O encode sentences using a BiLSTM [84]. We do not aim for parsers trained with ‘optimal’ hyperparameters but instead use (nearly) default/example training configurations provided in the corresponding GitHub repositories, as this is more aligned with real-world use cases. For example, the repository provides example training configurations for Biaffine and CRF2O, where the recommended Biaffine uses RoBERTa, while CRF2O uses BiLSTM. Moreover, optimization may further lead to overfitting on out-of-domain data, a particularly urgent issue in the diachronic setting we face.

### Tokenizers

Except for Stackpointer, which does not have a built-in tokenizer, and CoreNLP, which has its own tokenizer, the other parsers implement the tokenizer from Stanza by default. Thus, for ease of use and to focus more on the parser differences per se, we implement the Stanza tokenizer for every parser consistently, disabling sentence splitting, as we will apply them to the curated sentences from our target corpora, for which further sentence splitting is unexpected.

For the out-of-box parsers—CoreNLP, Stanza, and TowerParse—there are mismatches between the training data and the evaluation data used later, both in terms of selected treebanks and UD versions. Besides, the parsers leverage different pre-trained models, which naturally introduces a performance gap (e.g., CRF2O with BiLSTM vs. Biaffine with RoBERTa). Consequently, the performance differences observed in our evaluation reflect the performance of individual checkpoints rather than the overall superiority or inferiority of any parser.

### 4.1 Evaluation

To explore the reliability of the parsers used, we evaluate them on (1) existing **UD treebanks**, (2) the treebanks built upon the two target corpora of this work (denoted as “**Target Treebanks**”), and (3) adversarially attacked treebanks concerning the most severe issues identified in §3. Table 4 provides the sizes of the treebanks in columns ‘Test’, ‘Target’ and ‘Adversarial’ for (1), (2), and (3), respectively.

Typological asymmetries between English and German could plausibly shape parser error profiles and, in turn, bias particular metrics. German’s richer inflectional morphology (e.g., case) and greater word-order variability (V2 in main clauses and frequent verb-final order in subordinate clauses) can increase ambiguity in attachment and long-distance dependencies, whereas English’s more rigid constituent order provides stronger positional cues but may concentrate errors in different constructions.

### Metrics

We calculate the **Unlabeled Attachment Score (UAS)** and **Labeled Attachment Score (LAS)** for the parser performance. UAS and LAS are originally defined as the percentage of tokens with correctly predicted heads and head+relations respectively; in this scenario, the input of the parser is sentences pre-tokenized by humans. On the other hand, [85] test parser performance in a real-world scenario, i.e., parsing the raw text without any gold standard (e.g., sentence segmentation, tokenization, part-of-speech tags, etc.). The metrics are then re-defined as the harmonic mean (F1) of precision P and recall R (using LAS as an example):


$$P=\frac{\#correctRelations}{\#predictedTokens}$$ (1)

$$R=\frac{\#correctRelations}{\#goldTokens}$$ (2)

$$LAS(F1)=\frac{2PR}{P+R}$$ (3)

where P is the percentage of the correct head (+relation) to the number of predicted tokens, while R is the percentage of the correct head (+relation) to the number of tokens in the gold standard. As we will use the parsers on sentences (without tokenization), we argue that *the re-defined metrics on raw texts are more indicative in our case*. For all evaluations, we adopt the evaluation script from the CoNLL18 dependency shared task [85], using sentences as input (without gold tokenization). This script ignores the subtypes of the relations (e.g., for relation ‘acl:relcl’ (relative clause modifier), only ‘acl’ is considered.), but here, we consider the full relations including the subtypes.

#### 4.1.1 Evaluation on UD treebanks.

To ensure the correctness of the training, implementation, and usage of the parsers, we first evaluate the parsers on the test sets of the existing UD treebanks, for which we choose Universal Dependencies (UD) v2.12, and compare the results to [85]. We exclude the treebank “GUMReddit” for English, as there are many nonsensical annotations. The treebanks for evaluation then include Atis, ESLSpok, EWT, GENTLE, GUM, LinES, PUD, ParTUT, and Pronouns for English (9 treebanks), and HDT, GSD, PUD, and LIT for German (4 treebanks). Although our evaluation setup, parsers, and the version of the UD treebanks are different from theirs, comparable (or better) results are expected since (1) the discrepancy in UD versions is small (mostly about fixing a tiny portion of incorrect annotations, e.g., see change logs in the official GitHub repository of UD treebanks, (2) most of the tested parsers are published later than the shared task and (3) the parsers do not need to split the text into sentences by themselves here.

## Results

In § A.1 in S1 Appendix, we confirm the legitimacy of the used parsers by showing that the performance of our used parsers is mostly superior to those in [85]. Next, we show the macro average and the standard deviation of the UAS and LAS over the UD treebanks in Table 5 (column “**UD**”). Biaffine consistently outperforms the others across different languages in terms of both UAS (en: 92% vs. 79%–88%; de: 87% vs. 79%–86%) and LAS (en: 89% vs. 73%–85%; de: 82% vs. 70%–80%). Furthermore, it also exhibits the strongest robustness across various treebanks, as demonstrated by the smallest average standard deviation (∼2.55 percentage points (**pp**)) over different metrics and languages. It is noteworthy that CoreNLP, the parser leveraged by all other related approaches, is the worst in our evaluation, according to all criteria.

**Table 5:** UAS and LAS on UD and Target treebanks. We show the macro average and the standard deviation of the metrics over the UD treebanks. The best performance in each criterion is bold and the second best one is underlined. We mark the parsers trained on mismatched data (with respect to UD versions or treebanks) with ^*^. Values in brackets indicate the absolute differences.

	UAS		LAS	
	UD	TARGET	UD	TARGET
English				
CoreNLP*	78.6 ± 5.5	82.9 (+4.3)	73.4 ± 6.5	78.7 (+5.3)
Stanza*	88.2 ± 6.2	88.0 (−0.2)	84.9 ± 8.0	85.0 (+0.1)
TowerParse*	87.1 ± 4.5	**92.8 (+5.7)**	82.8 ± 5.9	**90.3 (+7.5)**
StackPointer	85.7 ± 5.2	84.2 (−1.5)	81.3 ± 6.2	80.2 (−1.1)
CRF2O	85.0 ± 4.7	84.4 (−0.7)	80.5 ± 5.6	79.8 (−0.7)
Biaffine	**91.6 ± 2.6**	90.1 (−1.5)	**88.5 ± 3.3**	87.2 (−1.3)
(German)				
CoreNLP*	74.0 ± 2.9	74.4 (+0.4)	67.3 ± 2.9	69.6 (+2.3)
Stanza*	84.3 ± 4.1	87.6 (+3.3)	78.8 ± 4.4	82.0 (+3.2)
TowerParse*	86.2 ± 2.0	89.7 (+3.5)	80.3 ± 1.3	84.5 (+4.2)
StackPointer	83.6 ± 1.3	86.9 (+3.3)	78.2 ± 1.6	80.8 (+2.6)
CRF2O	82.1 ± 1.5	85.8 (+3.7)	75.1 ± 2.5	78.6 (+3.5)
Biaffine	**87.0 ± 2.5**	**90.8 (+3.8)**	**81.7 ± 1.8**	**84.5 (+2.8)**

Stanza trained on slightly mismatched data outperforms the other fine-tuned parsers on average for English. Nevertheless, it is the least robust one according to its high standard deviations (6.2 pp vs. < 5.5 pp in UAS; 8.0 pp vs. < 6.5 pp in LAS). Interestingly, Towerparse, which was trained on mismatched data in terms of treebanks and versions, performs similarly to Biaffine on German treebanks, only ∼1 pp lower UAS/LAS. However, we observe that it tends to predict multiple roots for one sentence and produce cycles in the dependency tree graphs, violating the UD dependency definition. In our evaluation, it generates 440 multi-root predictions and 1,498 cycles, while the others rarely make such errors (only Stackpointer predicts multiple roots 28 times among the other parsers). Besides, it also has to skip 28 sentences due to the limitation of its implementation (see the authors’ explanation about skipping sentences in footnote 7 in [79]).

### 4.1.2 Evaluation on target treebanks.

We leverage the dependency annotations from [86], which collects human annotations by manually correcting the automatic parsing results made by the Biaffine or CRF(2O) parsers from the SuPar toolkit (with their own trained checkpoints); it includes annotations in UD format for 111 sentences from Hansard and 163 from DeuParl from different time periods. According to the original paper, only the OCR errors in the sentences were corrected before parsing; therefore, this treebank may not fully represent the target corpora, but the domain-specific language style and usage persist in the treebank. There were two annotators involved, who annotated independently and in case of disagreement resolved them. Noting that the correction procedure may introduce bias in annotations due to the “priming effect” [87], they conduct further analysis by annotating a portion of the sentences from scratch and comparing the outcomes to those obtained through correction. The results suggest that humans are indeed biased due to the correction procedure. Nonetheless, they point out that some differences in annotations stem from inherent ambiguities, such as how multi-token proper names should be handled.

## Results

We show the evaluation results on the Target treebank in Table 5 (column “**Target**”). The values in the brackets indicate the absolute difference in metrics between the Target treebank and the UD treebanks. We observe that the Target treebank mostly does not negatively affect the parsers’ performance. All parsers exhibit improved performance on the **German** Target treebank compared to the UD treebank, with an increase ranging from 0.4 to 4.2 pp. For **English**, the maximal performance drop is observed with StackPointer and Biaffine (1.1–1.5 pp), while CoreNLP and TowerParse achieve better results, with an increase ranging from 4.3 to 7.5 pp. Stanza and CRF2O display only minor fluctuations (<1 pp). Biaffine and CRF2O may benefit from the gold standard collection method because some annotations were obtained by correcting outputs from parsers with the same architectures. However, our results suggest that parser performance on the target treebank is highly correlated with their performance on the UD treebank, shown with a Pearson of 0.875 for LAS. Consequently, the impact from this factor is minimal. However, as the OCR errors appear frequently in the German corpus but were excluded from the target treebank, we further investigate the impact of data noise below.

### 4.1.3 Evaluation on adversarial treebanks.

To further inspect the effect of data noise on the parsers, we generate two adversarial datasets concerning the two most prevalent issues in German data identified in §3, i.e., historical spelling and OCR-raised spelling errors. We use the instances from the UD test sets as candidates for perturbation. To perform the historical spelling attack, we chose 23 candidate words from our previous annotations and replaced them with their historical spellings in the candidates sentences. This resulted in 1,948 sentences that were affected by the attack. For (2), we randomly replace 10%, 30%, or 50% of the characters in 1 or 2 tokens within a sentence with random characters, resulting in 6 attacking levels; we produce 2000 sentences per attack level.

### Results

**Fig 6:**
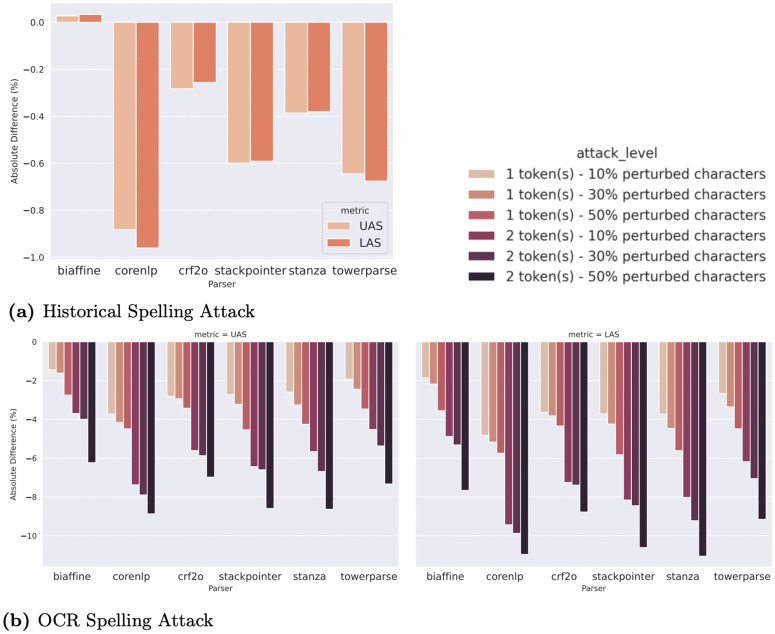
Absolute difference in UAS and LAS between the sentences before and after the attack.

We illustrate the absolute difference in metrics between the sentences before and after attacking in Fig 6, where y-axes display the differences in percent points (**pp**). Both types of attacks negatively impact parser performance, with LAS consistently being more affected compared to UAS. This can be explained by the fact that LAS is more sensitive to perturbations because it considers both attachment and labeling accuracy, whereas UAS only considers attachment accuracy. Even if the attachment remains correct, changes to the words themselves (such as spelling or character replacement) can result in incorrect labels. The only exception is the Biaffine parser, where the **historical spelling attack** does not degrade its performance but surprisingly boosts it slightly. However, the change in LAS/UAS is very small (<0.03 pp), which may be due to random variation, as depicted in Fig 6a. The performance of the other parsers is weakened by a decrease in UAS of ∼0.2–0.9 pp and in LAS of ∼0.2–0.95 pp, with CRF2O being the most robust and CoreNLP the least.

We present the results for **OCR spelling attack** in Fig 6b. OCR spelling errors have a much larger impact compared to the historical spelling changes, degrading parser performance up to around 11 pp in LAS and 9 pp in UAS, when 50% characters of 2 tokens within a sentence are perturbed. Again, Biaffine is the most robust against OCR attack. At the highest attack level, CoreNLP, StackPointer, and Stanza show similar sensitivity. However, at low attack levels, StackPointer and Stanza demonstrate better robustness compared to CoreNLP. In real-world cases, OCR spelling errors often involve only one character within a sentence. For example, in the sentence “... wollen *Tie* nun das letzte nehmen.” (from our annotation in §3), the only OCR error is in “*Tie*”, which should be corrected to “*Sie*”. Hence, Stanza and StackPointer are still superior to CoreNLP in real-world scenarios.

### Summary

Among the parsers, Biaffine shows the best performance in standard evaluations as well as the highest robustness under adversarial conditions. CoreNLP is often the worst one in our evaluation; besides, it is unable to predict crossing edges, which we consider to be an important indicator of language change. Moreover, the parsers seem to be sensitive to OCR errors in the data. Nevertheless, this does not necessarily mean they cannot be used in further analysis. As long as the diachronic trends in these metrics can be accurately observed and maintained in the presence of data noise, the parsers are reliable for further analysis; we explore this below.

#### 4.1.4 Sensitivity of metrics to data noise.

Trends are based on the rankings (and scales) of data points in a time series, so it is crucial that these rankings are not affected by data noise. To evaluate in a real-world scenario, we leverage the annotations collected in §3, where problematic sentences are flagged and paired with human-made corrections. We parse all sentences in the annotations (including perfect ones) before and after correction and calculate the 15 metrics based on their dependency trees. In § A.2 in S1 Appendix, we demonstrate that our metrics are insensitive to data noise by showing that the metrics for the clean data are highly correlated with those for the noisy data (overall 0.970 Spearman).

## 5 Metrics

Unlike most of the relevant approaches that only focus on the linear dependency distance [6,11–13], we examine a large array of metrics that capture statistical patterns driven by graph theory or linguistic phenomena. In the following, we outline the definition and usage of these metrics over a German sentence—*Das hat alles sehr gut geklappt!* (‘This has all worked out very well’) (extracted from the UD GSD treebank).

### Root Distance (*d*_*root*_)

**Fig 7:**
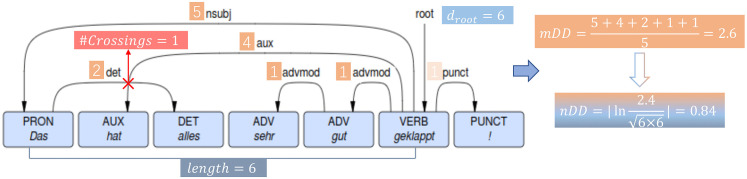
Illustration of observing *d*_*root*_ and *#Crossings*, along with calculating *nDD* and *mDD* using equation 4 and 5, with the example sentence “Das hat alles sehr gut geklappt!”. Arrows point from head to dependent tokens. Values with an orange background indicate the dependency distance between a dependent-head pair.

*d*_*root*_ is the index of the word connecting to the pseudo root in a sentence. An example is shown in Fig. 7, where *d*_*root*_ is 6 (from root to ‘geklappt’). [58] consider *d*_*root*_ as a normalizing factor when computing the mean of dependency distances for all dependency pairs in a sentence. [12] show that *d*_*root*_ correlates positively with dependency distance over time in English; specifically, *d*_*root*_ increases over time for long sentences while decreasing over time for short sentences.

### Mean Dependency Distance (*mDD*) and Normalized Mean Dependency Distance (*nDD*)

Following [11,13,58], we consider two statistical features of dependency distances, namely *mDD* and *nDD* given by:


$$\text{mDD}=\frac{1}{n}\sum_{(i,j)\in D}|i-j|$$ (4)

$$\text{nDD}=\left|\log\left(\frac{mDD}{\sqrt{d_{root}\times \text{\{length}}}\}\right)\right|$$ (5)

where |i−j| represents the absolute difference between the position indices of a dependency pair (*i*,*j*), *D* is a set of dependency pairs in a sentence (punctuation and root dependencies are excluded), length is the number of words in a sentence, and *n* is the size *D*. In Fig 7, *mDD* and *nDD* are 2.6 and 0.84 for example.

### Number of crossings (*#Crossings*)

**Fig 8:**
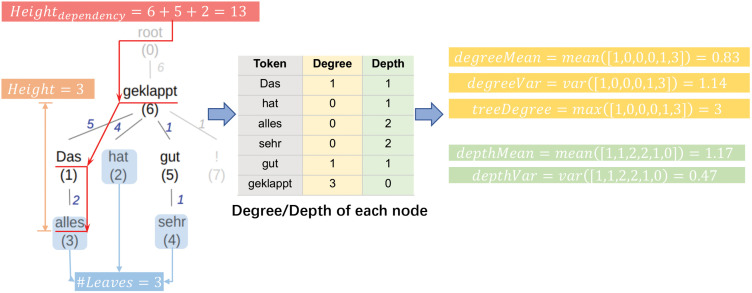
Illustration of calculating/observing *Height*, *Height_dependency_*, *#Leaves*, *treeDegree*, *degreeMean*, *degreeVar*, *depthMean* and *depth Var* with the example sentence “Das hat alles sehr gut geklappt!”. Values in brackets are the position indices of the tokens. Values beside edges indicate the dependency distance of the corresponding dependency pairs.

A crossing is said to be observed when two dependency relations overlap. [88] show that *#Crossings* correlates positively with dependency distance in 21 out of 30 languages, while [50] find that machine-generated artificial texts without crossings produce shorter dependency distances compared to those with crossings. We count the number of crossings in a sentence, ignoring punctuations. An example is shown in Fig. 8: the dependency relation between “Das” and “alles” crosses over the relation between “geklappt” and “hat”, i.e., *#Crossings* = 1.

### Number of leaves (*#Leaves*)

We count the number of leaves (*#Leaves*) of a dependency tree, excluding punctuations—see an example in Fig 8 (left). Intuitively, a tree with more leaves is wider and thus exhibits lower hierarchical levels.

### Tree height (*Height*) and Longest path distance (*Height*_*dependency*_)

Dependency tree depth/height is seen as an indicator of syntactic complexity [89,90]. In this work, we define two metrics based on that: *Height* is the longest-path distance from the root node to a leaf, while *Height*_*dependency*_ is the weighted longest-path distance where an edge over two nodes is weighted by the dependence distance between the nodes. An example can be observed in Fig 8 (left): *Height* = 3, *Height*_*dependency*_ = 13.

### Depth variance (*depthVar*) and Depth mean (*depthMean*)

We denote the depth of a node as the path length from the node to the root. In addition to [89,90], who only considered tree depth (i.e., the maximal node depth across all nodes in a tree) to measure syntactic complexity, we also consider the mean and variance of node depths, denoted by *depthMean* and *depthVar*, respectively. The calculation example is illustrated in Fig 8 (middle).

### Tree degree (*treeDegree*) and Degree variance (*degreeVar*) and Degree mean (*degreeMean*)

As shown in Fig 8 (middle), we measure the degree of a node as the number of outgoing nodes attached to that node, i.e., by counting the dependents of a head word in a dependency tree. The higher the degree of a node, the more dependents a head word has. This metric is related to dependency distance—as both long dependency distances and head words with high degrees can complicate the syntactic structure, in line with the use of branching factor as a syntactic complexity measure in [89,90]. We also consider statistical features: the mean (*degreeMean*) and variance (*degreeVar*) of the degrees of all nodes, as well as the maximum of all nodes’ degrees (*treeDegree*).

### Head-final Ratio (*Ratio*_*head-final*_) and Head-final Distance (*d*_*head-final*_)

**Fig 9:**
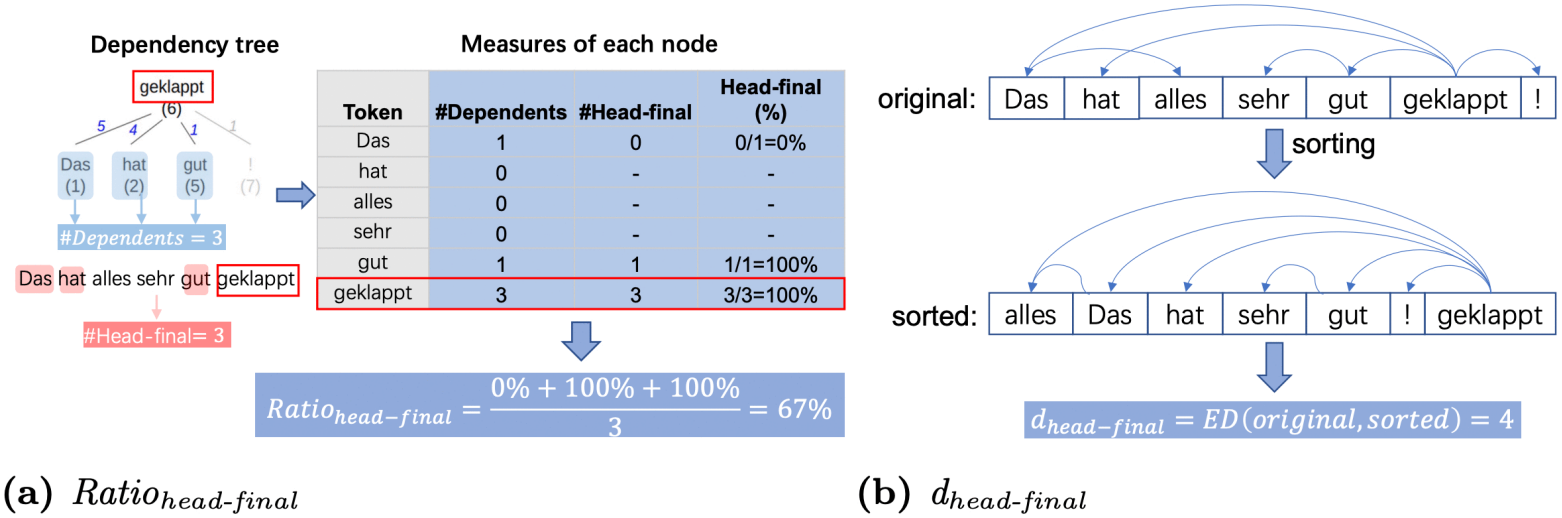
Illustration of calculating/observing *Ratio_head-final_* and *d_head-final_* with the example sentence *Das hat alles sehr gut geklappt!.* Arrows point from head to dependent tokens.

For a dependency pair, if a head follows its dependent, then the pair is termed as a head-final pair. Otherwise, if a head precedes its dependents, then the pair is considered head-initial. In linguistics (language typology), this head directionality is profiled as a crucial parameter for classifying languages [91]. In Fig 9a, for each head in a sentence, we calculate the percentage of its head-final pairs to all pairs with that head (head-final(%)), and then average the head-final(%) over all heads in that sentence, termed as *Ratio*_*head-final*_. We note that the sentence *Das hat alles sehr gut geklappt!* not only includes head-final dependency pairs, e.g., *geklappt* following its dependents *das*, *hat* and *gut*, but also head-initial pairs, e.g., the head *das* preceding its dependent *alles*. Here, we consider how distant this sentence is, with a mix of head-initial and head-final pairs, compared to the same sentence but permuted to have only head-final pairs. This results in the metric *d*_*head-final*_ that is the Levenshtein edit distance [75] between the original and permuted sentences, as illustrated in Fig 9b.

### Random tree distance (*d*_*randomTree*_)

Inspired by [5,8], who check whether dependency distances in human language are shorter than chance when investigating DDM, we inspect whether the general dependency tree structure of human language has evolved in a certain direction. To do so, we randomly permute the dependency tree structure in a sentence and then compute the tree edit distance between the original and permuted trees using the algorithm of [92].

## 6 Analysis

Following [6,12], we leverage the Mann Kendall (MK) trend test [93] to indicate the diachronic change of the concerned metrics. The MK trend test is widely used to detect trends in time series. It has three outputs: “increasing”, “decreasing”, and “no trend”; an increasing or decreasing trend is detected only if it is significant with a p-value <0.05. We perform it for the 9 sentence lengths and 15 metrics, totalling 15 × 9 = 135 test cases.

We first explore the dependence of the trends on parsers in §6.1 to answer RQ2 (*Can one rely on the predictions of a single parser?*), as all of the relevant works employed a single-parser approach [e.g., 6, 12]. Then, we compare the syntactic changes between English and German in the remaining sections, using the proposed syntax metrics, to answer RQ3 (*Do English and German mostly change similarly or differently?*) and RQ4 (*How do English and German change when looking at syntactic dependency graph properties beyond dependency distances?*). Specifically, we inspect the overall similarity of syntactic changes between English and German in §6.2, with a focus on sentence length in §6.3. Next, we analyze different and similar trends between the two languages in §6.4 and §6.5 respectively.

### 6.1 Can one rely on the predictions of a single parser?

**Fig 10:**
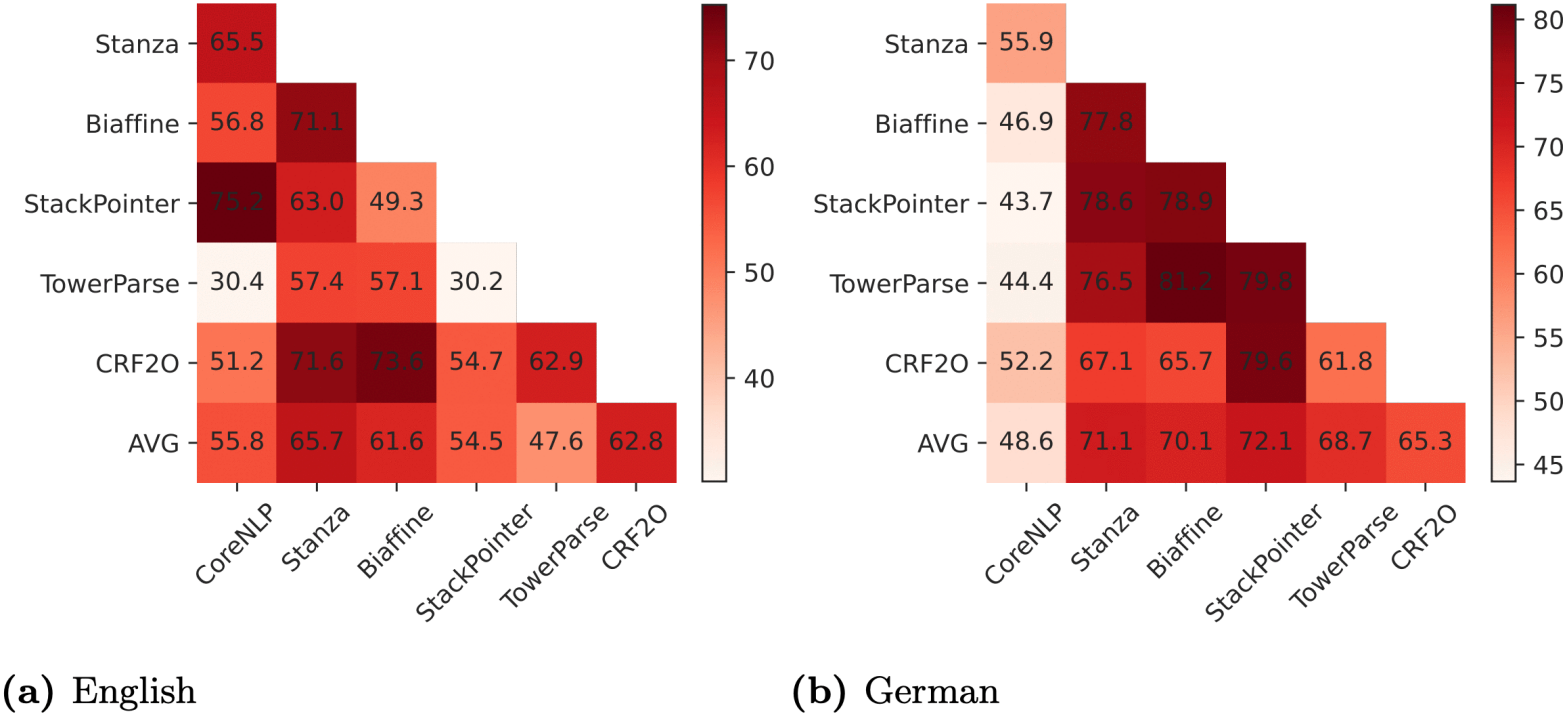
Cohen’s Kappa of MK results based on the dependency relations predicted by different parsers. Deeper colors denote higher agreement and vice versa. We show the average agreement for each parser in the last rows.

We first inspect how the parsers agree with each other on the predicted diachronic syntactic trends based on the MK outputs. The outputs of MK can be seen as the labels of a three-way classification task. Therefore, we calculate Cohen’s Kappa [74] to indicate the agreement between each parser pair. The values in Fig. 10 are derived by calculating Cohen’s Kappa across the 135 MK outputs based on different parsers for each language. We then average the agreements over all parser pairs containing a certain parser to achieve the average agreement for each parser, shown in the last rows in Fig. 10.

As illustrated in Fig 10a, on **English** data, most parsers demonstrate a moderate agreement with others (0.4–0.6); the highest agreement of 0.752 is observed between CoreNLP and StackPointer, followed by Biaffine and CRF2O (0.736). Among the parser pairs, TowerParse and StackPointer obtain the lowest agreement of 0.302. Stanza obtains the highest average agreement with others (0.657), whereas TowerParse has the lowest one (0.476). For **German** (Fig 10b), in contrast to English, most parsers obtain a substantial agreement with others (0.6–0.8). The highest agreement is achieved between Biaffine and TowerParse (0.812), followed by TowerParse and StackPointer (0.798); StackPointer achieves the highest average agreement (0.721), while CoreNLP least agrees with others on German data (0.437–0.559). Our observations indicate that **the dependency parsers often disagree with each other in terms of the predicted trends.** The German parsers in our experiments may benefit from the more consistent training data because of the dominance of the HDT treebank, which makes up a substantially larger proportion of the German training data compared to the biggest treebank (EWT) in the English training data (91% vs. 38%). Besides, the German training data is around 4 times larger than the English one (166,849 vs. 32,865 sentences). Therefore, German parsers may have a higher chance to achieve higher agreements; however, even with identical training data, different parsers (StackPointer, Biaffine, and CRF2O) still yield varying trend predictions. This raises concerns about previous approaches which solely rely on one parser.

As we cannot automatically determine which parser produces more reliable trend predictions, we leverage the ensemble of these parsers to predict the most likely correct trends (cf. ‘wisdom of the crowd’ [94]). Specifically, we employ a **majority vote** approach: if the increasing/decreasing trends can be detected based on at least 4 out of 6 parsers, we consider them valid; an exception is made for *#Crossings*, where we relax the threshold to 3 out of 6 due to CoreNLP’s inability to predict crossing dependencies. Since our main goal is to compare English and German, we also examine the differences in the number of same/different trends between the two languages across individual parsers and the ensemble parser. As shown in Table 6, *different parsers exhibit distinct patterns*. For instance, CoreNLP and TowerParse miss more than one third of the same trends predicted by the ensemble parser (–12/–11 vs. 29), whereas Biaffine and StackPointer predict approximately 34% and 55% more same trends (+10/ + 16 vs. 29), respectively.

**Table 6:** Differences in the number of predicted same/different trends between English and German across the ensemble and single parsers. The ‘Ensemble’ column shows the total number of same/different trends between English and German predicted by the ensemble parser. For the other columns, the first entry (+) indicates the number of additional trends predicted by a given parser relative to the ensemble parser, and the second entry (–) indicates the number of missed trends relative to the ensemble parser.

Trends	Ensemble	CoreNLP	Stanza	Biaffine	StackPointer	Towerparse	CRF2O
Same	29	+7 | −12	+4 | −5	+10 | −3	+16 | −2	+2 | −11	+7 | −1
Different	3	+2 | −1	+4 | −0	+1 | −0	+3 | −0	+3 | −0	+0 | −1

**Answer**: The observations above imply that **one cannot rely on a single parser** for such studies, as the parsers often disagree with each other and there can be substantial differences in the observed diachronic trends when using the ensemble parser versus individual parsers.

Therefore, to increase the likelihood of correctness, we base our analysis on the results from the ensemble parser. The final trends for the 135 cases based on the ensemble parser are listed in Table 8 in S1 Appendix.

### 6.2 Are syntactic changes more alike or distinct in English and German?

**Fig 11:**
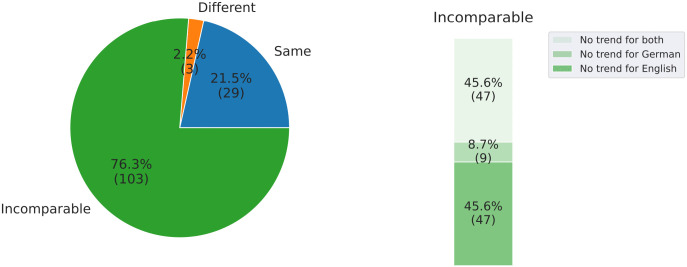
Left: Percentages of the same, different and incomparable trends between English and German. Right: Percentages representing incomparable trends due to ‘no trend for English,’ ‘no trend for German,’ and ‘no trend for both’ to all incomparable trends. Values in brackets indicate the counts of trends.

We compare the 135 trends between English and German for the 15 metrics and 9 sentence lengths. As shown in Fig 11 (left), among all the trends, 103 cases (76.3%) are incomparable since there are no significant trends for German or English or both; 29 cases (21.5%) have the same trends, while only 3 cases (2.2%) show different trends, which may suggest **a more uniform (“convergence”), rather than diverse, syntactic change between English and German**. Among the incomparable trends, as shown in Fig 11 (right), 47 cases (45.6%) are the result of no significant trends for both English and German, 9 cases (8.7%) lack significant trends for German, and 47 cases (45.6%) have no significant trends for English. From this perspective, the German language has undergone more significant syntactic changes compared to English.

**Answer**: The results suggest that syntactic changes are **more alike** in English and German rather than more distinct.

### 6.3 Which sentence lengths exhibit the most distinct or similar syntactic changes between English and German?

**Fig 12:**
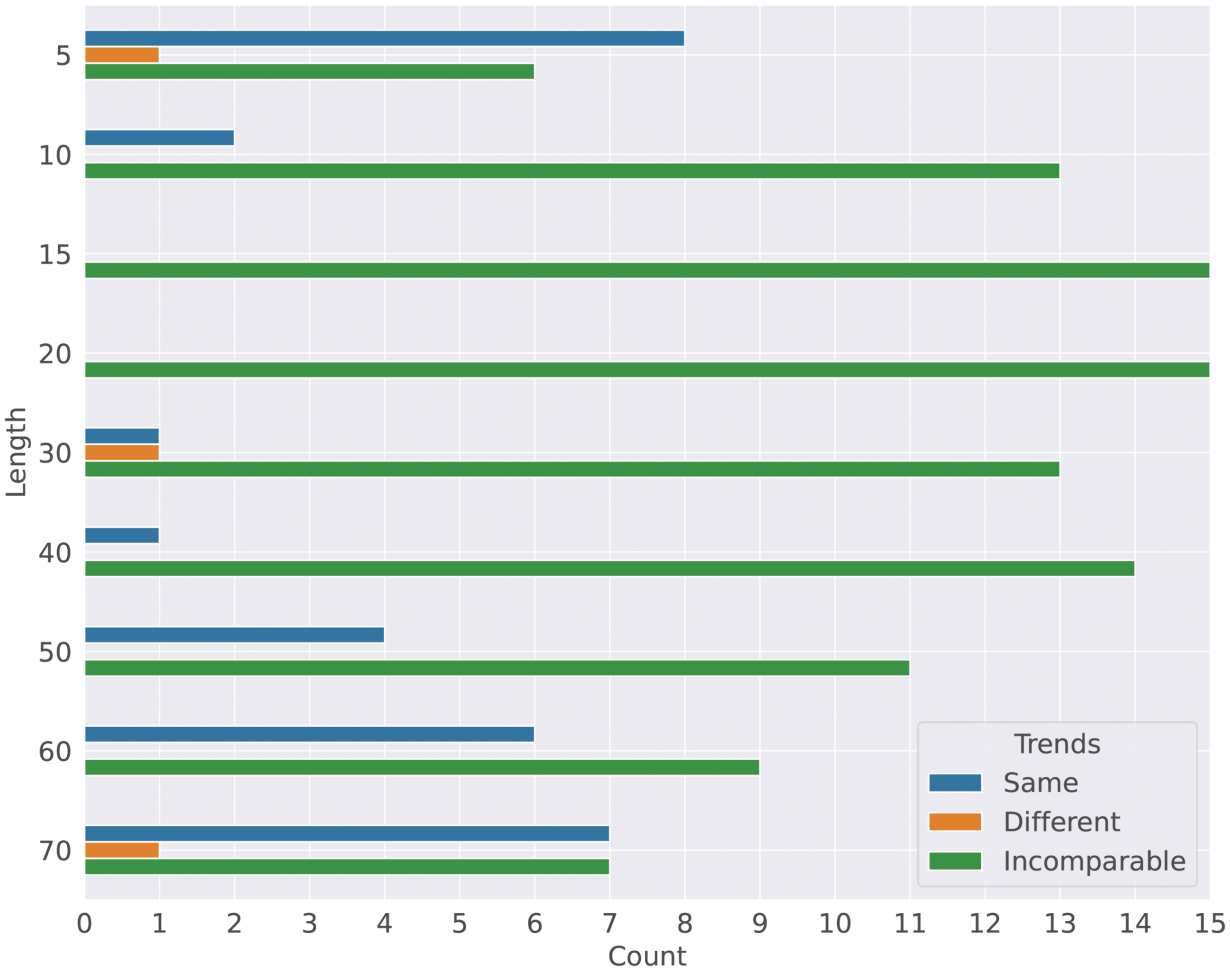
Number of same/different/incomparable trends for each sentence length group. Larger numbers indicate more metrics exhibiting the same/different/incomparable trends between English and German for a specific sentence length group.

We count the number of metrics showing different/same/incomparable trends between English and German for each sentence length, displayed in Fig 12. We observe that: (1) for sentences of lengths 5, 30, and 70, 1 out of 15 metrics exhibits different trends, whereas there are no different trends for the other lengths; (2) *sentences of length 5–7 behave most similarly*, shown with the highest number of metrics with the same trends (8 out of 15) among sentence lengths, followed by the longest sentences of length 70–72, which have 7 out of 15 metrics with the same trends; (3) the number of metrics with the same trends increases from 1 to 7 as sentence lengths go from 30 to 70. We also note that the distribution of the incomparable trends (green bars) seem to follow a Gaussian distribution, where there are more incomparable trends for the sentences of middle lengths (e.g., 15 and 20), while less incomparable trends exist for the shortest and longest sentences. Interestingly, as we show in Fig 18 in S1 Appendix, sentences of middle lengths dominate in the corpora; this may suggest that syntactic variation is more likely to occur in sentences of extreme lengths (i.e., longer and shorter sentences) rather than in sentences of common lengths.

**Answer**: In summary, sentences of **extremely short or long lengths** (lengths 5 and 70) exhibit **most similar** syntactic changes between English and German, whereas no clear evidence emerges regarding the sentences of certain lengths SE: regarding the lengths showing distinct changes? Don’t understand this part. I was expecting middle range sentence lengths, given that short and long sentences were just now discussed showing most distinct changes, as there are only three distinct trends, which are evenly distributed across all lengths examined.

### 6.4 What are the different trends in syntactic changes between English and German?

**Fig 13:**
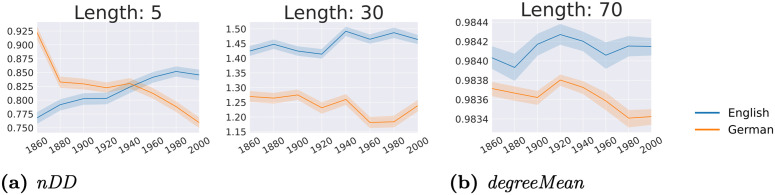
Metrics showing different diachronic trends based on MK between English (blue) and German (orange), averaged per decade group and over the 6 parsers. We show 95% confidence intervals.

**Answer**: **3 distinct diachronic trends for 2 metrics between English and German** are observed, as visualized in Fig 13. It shows the metrics for English (blue) and German (orange) over time, averaged over the 6 parsers per decade group. For ***nDD*** (Fig 13a), the trend goes up for English but down for German across both sentence lengths. Fig 13b shows the trends of ***degreeMean*** for sentences of length 70–72. Overall, while *degreeMean* increases for English over time, it decreases for German, suggesting that, on average, words govern more dependents over time for long sentences in English but fewer in German.

An interesting observation when closely examining the three different trend patterns in Fig 13 is that for two out of three cases, subtrends look quite similar across English and German. Witness *degreeMean* as a point in case; there is a sequence of four subtrends: downward, upward, downward, upward in both English and German, where the German pattern seems to lag behind compared to the English one (i.e., German seems to mimic the English pattern). This is a limitation of our overall trend analysis which takes a global pattern into account, disregarding local subpatterns (cf. Simpson’s paradox [95]).

### 6.5 What are the similar trends in syntactic changes between English and German?

**Fig 14:**
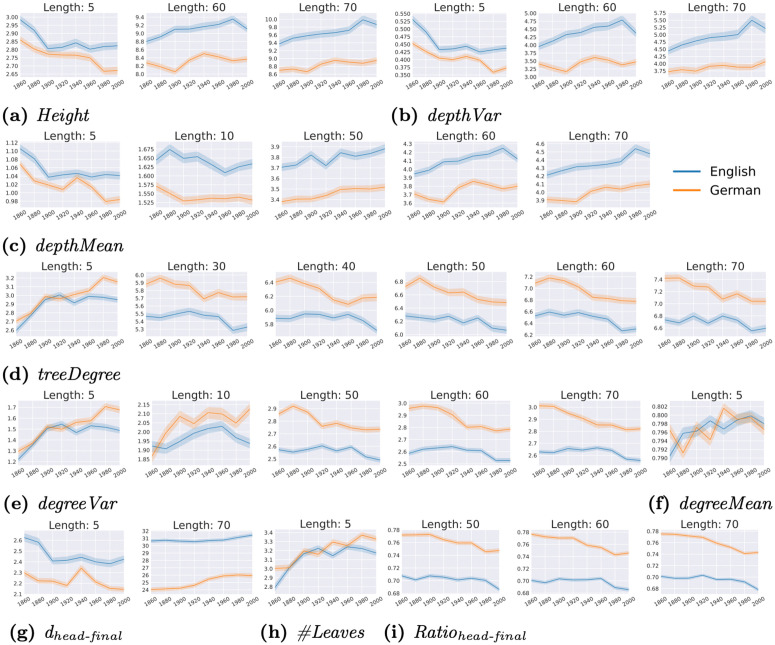
Metrics showing the same diachronic trends based on MK between English (blue) and German (orange), averaged per decade group and over the 6 parsers. We show 95% confidence intervals.

**Answer**: We identify **29 trends for 9 metrics that demonstrate simultaneous increase or decrease in both English and German**. We show the average of those metrics per decade group over the 6 parsers in Fig 14; blue lines represent the trends for English and orange lines for German.

**Fig 15:**
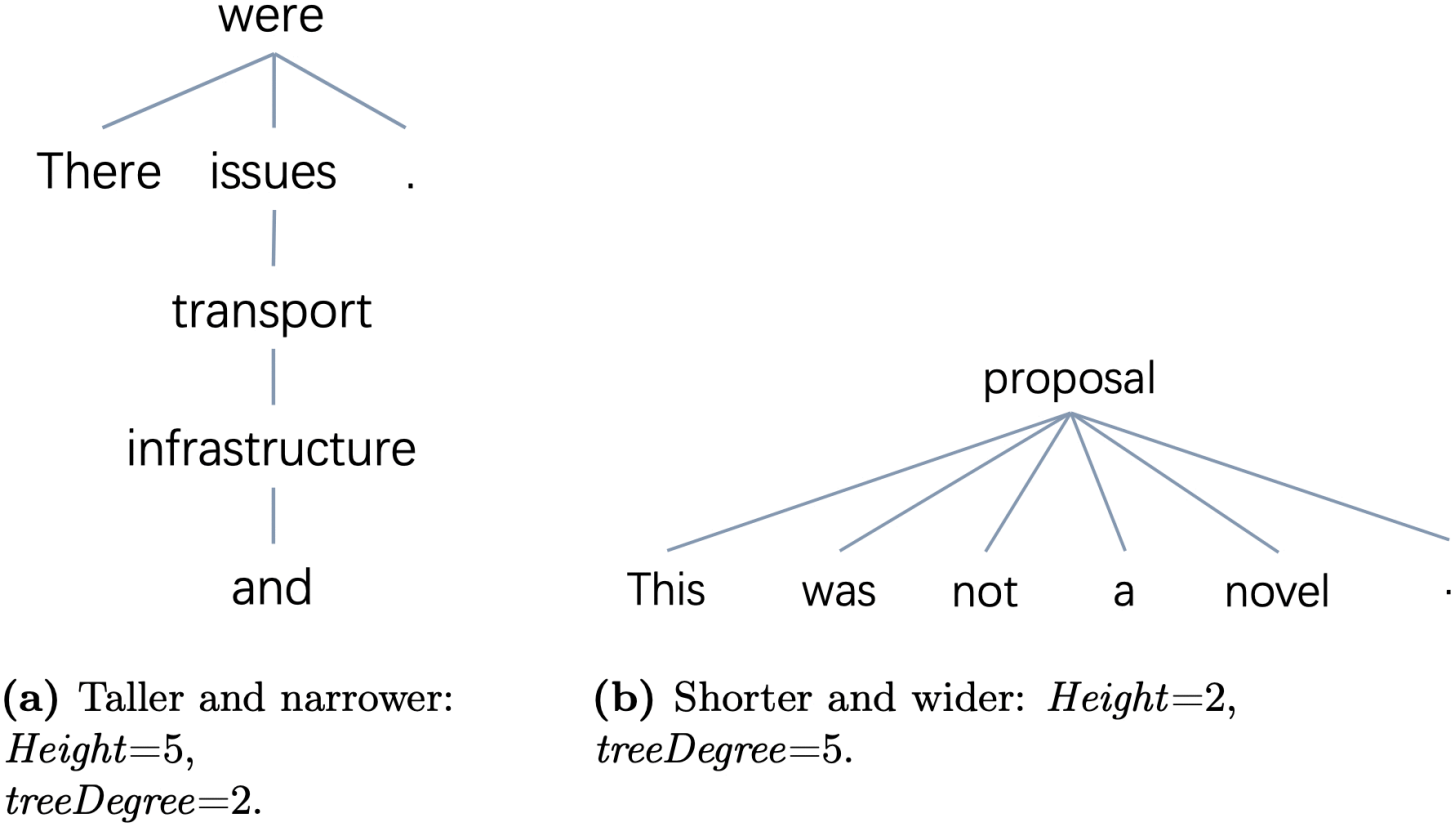
Dependency trees for sentences of length 7: taller and narrower vs. shorter and wider. Predicted by Stanza. Both sentences taken from the Hansard corpus: left: ‘There were transport and infrastructure issues.’; right: ‘This was not a novel proposal.’.

For the metrics assessing dependency trees vertically, namely ***Height*** (Fig 14a), ***depthVar*** (Fig 14b) and ***depthMean*** (Fig 14c), the trends are similar: they decrease over time for short sentences having 5–7 and/or 10–12 words but increase for long sentences like those of length 60–62 and 70–72. In contrast, the degree-relevant metrics, which assess trees horizontally, increase for short sentences but decrease for long sentences: in Fig 14e, 14d and 14f, we observe an increase in ***degreeVar***, ***treeDegree***, and ***degreeMean*** for sentences having 5–7 and/or 10–12 words, as well as a decrease in both *treeDegree* and *degreeVar* for longer sentences of lengths from 50 to 70 words. All of the above suggests that generally, for both English and German, the dependency trees of shorter sentences with ≤10–15 words become shorter and wider over time, while those of longer sentences having ≥30 words become taller and narrower over time. Short and wider tree structures have a higher chance that a word governs multiple dependents compared to the taller and narrower ones and vice versa. An example for comparing between the shorter and wider vs. the taller and narrower dependency trees is given in Fig 15, where both sentences have 7 tokens.

The trends of ***dhead-final*** and ***Ratio***_***head-final***_ are illustrated in Fig 14g and 14i respectively. A larger *d*_*head-final*_ of a sentence indicates greater distance from the corresponding fully head-final sentence (where all dependency pairs of that sentence are head-final); thus, it suggests that the sentence is more head-initial. *d*_*head-final*_ is expected to rise with declined *Ratio*_*head-final*_ to reflect language becoming more head-initial and vice versa. We see that: (1) *d*_*head-final*_ decreases for sentences with 5–7 words but increases for those having 70–72 words, implying that short sentences become more head-final, while long sentences become more head-initial; (2) *Ratio*_*head-final*_ decreases over time for sentences having ≥ 50 words, also suggesting a tendency for longer sentences being more head-initial over time.

Fig 14h displays the trends of ***#Leaves*** for sentences having 5–7 words, where we see an increase over time for both languages. This suggests a tendency that more words never serve as a head and a head governs more dependents in short sentences, which is consistent with the changes in overall dependency tree structures, i.e., they become shorter and wider for short sentences.

### 6.6 Summary

In this section, we addressed our remaining research questions from §1 with several key observations: (1) Observed trends in syntactic metrics heavily depend on the parsers used; there can be substantial differences between the predicted trends obtained from ensemble and individual parsers. Thus, when automatic verification of these predictions is not possible, one should not rely on a single parser for such studies (RQ2). (2) English and German mostly show convergent syntactic changes from 1860 to 2020 within the political speech domain (RQ3). (3) Sentences of middle lengths do not show an explicit syntactic change pattern. In contrast, we have seen clear but opposite syntactic change patterns for short and long sentences in both languages: Long sentences tend to have taller and narrower dependency trees over time, while the dependency trees of short sentences are becoming shorter and wider. Additionally, short sentences are trending towards head-final structures, whereas long sentences are becoming more head-initial (RQ4).

## 7 Discussion

### 7.1 Effect of sentence length

**Fig 16:**
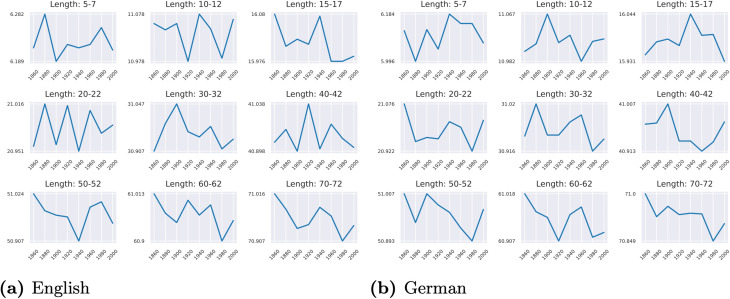
Average sentence length in our observation data over time. We show the maximal and minimal average lengths on the y-axes.

As mentioned in §3.2, it is important to control the sentence length for diachronic syntactic observations as it may be a confounding variable. Thus, we compute the diachronic trend of the average sentence length per decade group for each length group in our data, visualized in Fig 16. Even if the length difference within one group is up to 3 tokens in our data, we still often see a decrease in average sentence length over time, especially for the longer sentences with more than 50 tokens (last rows in Fig 16). However, the difference in the average sentence length within one length group is only up to ∼0.1 token, which we deem as an acceptable level. The reasons for this are the following: (1) Intuitively, such a small difference in sentence length is unlikely to influence our metrics substantially. For instance, it takes at least one additional token to increase the maximal possible *Height* and *treeDegree*. (2) We observe trends of metrics that counter the development of sentence lengths, e.g., that longer sentences have a higher chance to produce taller dependency trees compared to shorter sentences; however, we see uptrends in *Height* in Fig 14a for sentences with 60–70 words, whose lengths are overall decreasing over time for both languages. This suggests that such a minor decrease in sentence length does not substantially impact the observations on syntactic changes. (3) The subtrends in metrics do not often follow the subtrends in sentence length. For instance, in 6 out of 8 metrics in Fig 14 for sentences of length 5–7 (*Height*, *depthVar*, *depthMean*, *treeDegree*, *degreeVar*, *d*_*head-final*_), the corresponding trends behave consistently across both languages from the time window 1860–1870–1880–1890, even when German sentences become longer while English sentences become shorter during that period.

### 7.2 Scale of syntactic changes

We note that several of our syntactic trends, even if they are statistically significant, are small in scale. For example, *degreeMean* in Fig 13b changes from slightly above 0.9840 to slightly below 0.9842 in English over the past 160 years (not all trends are on such low scales, however: *d*_*head-final*_ increases from around 24–26 for sentences having 70–72 words (Fig 14g right), for example). On the one hand, this makes sense as syntax is expected to change much slower, e.g., compared to lexical or semantic changes. On the other hand, this may also mean that some of our identified changes may hardly be noticeable (by linguist experts) in actual language or will more pronouncedly manifest themselves only in the future.

### 7.3 Parser agreements based on mixed-effects regression

To see whether different trend prediction methods affect our observation that trend predictions vary across parsers, we also fit mixed-effects regressions for each metric and length group. We only keep the significant trends (p < 0.05) predicted by a converged regression model and treat all other outcomes as no trend predicted. As Table 9 in S1 Appendix shows, the agreements on predicted trends are even lower than those based on MK trend tests (English: 0.272–0.712 vs. 0.302–0.752; German: 0.409–0.749 vs. 0.437–0.812). English parsers obtain lower agreements than German ones, which is in line with our observations in §6.1. This further emphasizes that relying on a single parser for such a study is unreliable.

### 7.4 Generalizability to other domains

The analysis in this work is focused on parliamentary debates, raising the questions whether the observations also apply to texts from other domains. We therefore extend our analysis to a selection of different sub-domains: news articles, magazines, fiction, and academic works. Using the same sampling method as outlined in §3, we obtain sentences from the *Corpus of Historical American English* [COHA [96]] and the *Deutsches Textarchiv* [DTA [96]] corpora, with a slightly shifted time span of 1820–1960 compared to our parliamentary dataset. We conduct the same experiments discussed in §4 and §6 on the resulting corpus.

We observe that the mutual agreement of the parsers decreases strongly for both German and English, especially for German (0.28–0.44 vs. 0.49–0.72 average Cohen’s Kappa per parser for German and 0.45–0.64 vs. 0.48–0.66 for English); this strengthens our diagnosis of the influence of the underlying parser. The reason for these variations could be the more homogeneous function of parliamentary discourse compared to other domains: For instance, in the fictional domain, authors may aim to distinguish themselves, while the academic domain is internally diverse due to varying writing styles and terminologies across different research areas and subjects. Politicians who participate in parliamentary debates, on the other hand, are more concerned with manifesting their party affiliation, discussing a variety of topics from the same political perspective, and maintaining a speaking style that is oriented toward certain rhetorical traditions, which can make their speeches more similar than other texts from a syntactic point of view. In addition to the influence of genre, language dependency is also emphasized: the effects go in the same direction, but are stronger for German.

Because of the low agreement of parsers on German data, we cannot draw meaningful conclusions about language change from the majority vote results. However, for English, we are able to observe some similar trends to the parliamentary domain. For example, the long sentences are becoming taller and narrower, shown with the decreasing degree-relevant metrics and increasing height-relevant metrics. Besides, the *Ratio*_*head-final*_ is decreasing for sentences longer than 15 words, suggesting English is becoming more head-initial. The difference in syntactic changes between short and long sentences is not prominent in this domain though.

### 7.5 Linguistic interpretation of trends: Considerations on colloquialization

Although some previous studies have shown that the *mDD* is decreasing over time, in this work, we do not see direct evidence supporting this. In fact, *mDD* for German is increasing over time across 7 out of 9 lengths in our experiment, while it is only decreasing for English sentences having 70–72 tokens (see Table 8 in S1 Appendix). This raises the larger question of whether dependency distance optimization is indeed such an important driver behind syntactic change as some studies have suggested—linking dependency distance to complexity and communication efficiency—or whether other factors need to be considered as well.

Overall, our observations on *mDD* are more in line with [14] and [6], who base observations on the sentences of identical lengths instead of grouping sentences by a range of lengths [e.g., 11]. The former point to differences between text genres, mostly finding no trend or uptrend in *mDD* for ‘general’ text domains (as compared to scientific language), including news, magazines etc. (cf. also [50]). We observe that while our observed trends for *mDD* diverge from expectations based on the literature, some of the 14 other metrics our study considered also show interesting trends (as far as parsers agree), which raise the question what **linguistic properties** of parliamentary debates might have changed to be reflected in these trends. We would like to highlight in particular the fact that $d_{head-final}$ increases for long sentences, and that the trends for *degreeVar, treeDegree*, and *degreeMean* suggested that shorter sentences become shallower and wider over time, while longer sentences become taller and narrower. Compounded with the trends observed for *Height*, *depthVar*, and *depthMean*, indicating that shorter sentences become more shallow, and longer sentences become taller.

From the existing literature, we derive the hypothesis that changes in *mDD* may be related to changes in the use of complex nominal structures, as well as in paratactically organized structures [7]. Complex nominal structures with several adjectival and nominal modifiers, such as *temporary*_amod_ *visual*_amod_ *motion*_nmod_ *blindness* (example from [7]), are said to have a lower *mDD*, but are narrower and deeper. Paratactically organized sentences can be characterized by coordination or sequential apposition of main clauses, as opposed to hypotaxis, where a matrix clause contains embedded (complement and adverbial) clauses. The trends in our other metrics are consistent with that: The longer the sentences, i.e., the more words they contain, the more likely it becomes that complex nominal structures or hypotactic constructions are involved (leading to narrower and taller trees), while shorter sentences are prone to a more shallow organization. Equally, it is telling that $d_{head-final}$ increases in longer sentences: this points to exbraciation of material, i.e., moving it out of the core sentence and placing it at the end. This can be seen in the following example from DeuParl (2020), where *politisch...insgesamt* is placed after the sentence (which, as an embedded clause, ends with the finite verb *stellt* ‘puts’): *“... dass die Covid-19 -Pandemie das vereinte Europa nun endgültig vor seine bislang größte Bewährungsprobe stellt* ***—politisch, wirtschaftlich und nicht zuletzt auch hinsichtlich des Vertrauens in die Handlungsfähigkeit Europas insgesamt****”* (‘... that the Covid-19 pandemic is now finally putting the united Europe to its greatest test to date —politically, economically and, not least, in terms of confidence in Europe’s overall ability to act.’), instead of in the middle field, which would equally be possible: *“... dass die Covid-19 -Pandemie das vereinte Europa* ***politisch, wirtschaftlich und nicht zuletzt auch hinsichtlich des Vertrauens in die Handlungsfähigkeit Europas insgesamt*** *nun endgültig vor seine bislang größte Bewährungsprobe stellt”*. Exbraciation is more likely in conceptually oral registers [97,98]. In many cases in our corpus (such as the example), it may be related to information-structural reasons (focus), but such are of course processing-related, too [99].

It has been argued that there is a trend towards colloquialization in the parliamentary debates of some languages [65,66]. Although this has not yet been studied for German, and not for syntactic properties, we can interpret the trends in our metrics as being indicative of such an increasing colloquialization — a diachronic increase in **conceptual orality**. Traditionally, contributions to parliamentary debate are scripted and, for the most part, read out, thus reflecting a greater degree of **conceptually written** style. Over time, the syntax of parliamentary speeches gained more **conceptually oral** syntactic properties. For different degrees of conceptual and medial orality, we refer to the classification proposed by [61,63]. This is in line with a more general trend towards destandardization, as for German discussed in [100]. As such, this is not so much a systematic change affecting the language as a whole (as would be the shift from SOV to SVO between Old and Middle English, mentioned above), but rather a shift in the language norm, viz. a change in the distribution on systematically available options in the language [101]. The metrics presented in the current paper cannot as such distinguish between deeper systematic and more superficial syntactic changes in the language norm, but the fact that they can pick up changing distributions in (superficial) syntactic structures raises hopes for future work, especially if larger time depths and more genres were to be taken into account.

## 8 Conclusion

This study delved into diachronic language changes in English and German. We comprehensively examined 15 metrics, including the mean dependency distance, across extensive corpora containing political debates. By analyzing the agreement among six dependency parsers, including the commonly used Stanford CoreNLP, we provided novel insights into the importance of used parsers in such language change research. Notably, we discovered a distinctive uptrend in the mean dependency distance in German, which is consistent across various parsers and sentence lengths. We also found that German and English seemed to undergo similar syntactic changes in the two political corpora examined, with only few instances showing opposing trends. We also observed more syntactic diachronic variability in sentences of extreme lengths, e.g., below or above mean sentence lengths. Our results may be relevant in downstream tasks, e.g., for text generation systems that map across historic time frames, e.g., diachronic summarizers or MT systems [102].

Possible limitations of this work are the following: (1) The text genre mainly explored was restricted to political debates, in order to make the subcorpora comparable. As our discussion of the linguistic factors behind the observed trends indicates, though, this may be an important factor affecting the measures—parliamentary debates may behave differently in this respect from scientific texts as discussed in [7], and their behavior may be subject to diachronic shifts in the language norm. (2) The time depth of the used diachronic corpora only covers the last 160 years—which may be too short to draw conclusions about syntactic change, which tends to take several centuries to unfold [e.g., 103]. (3) We did not thoroughly investigate the origins of the changes in measures. For instance, [7] indicate that the downtrend in dependency distance over time is because dependency relations with shorter distances appear more frequently in the later periods and vice versa, while [12] show that longer sentences have more dependency relations with decreasing dependency distances compared to the short ones, which results in the discrepancy in the trends for short and long sentences.

Our study has been largely formal—investigating the role of parsers and metrics on the identification of language change in English and German—but remains preliminary regarding the detection of actual syntactic change, and identifying its causes. Future work should complement our analysis in this respect.

## Supporting information

S1 Appendix A. Spearman correlation, annotation guidelines, sentence length distribution, parser LAS scores, metrics that have a stable trend supported by at least 3 parsers, and Cohen’s Kappa of predicted trends based on mixed-effects regression. (PDF)
