# Supplementary material for: Syntactic language change in English and German: Metrics, parsers, and convergences
Source: PLoS One. 2026 Apr 28;21(4):e0346096. doi: 10.1371/journal.pone.0346096 (PMC13123979; doi:10.1371/journal.pone.0346096)
Supplement: S1 Appendix — (PDF) [file pone.0346096.s001.pdf]

A Appendix

A.1 Comparison to Zeman et al. [85]

As Fig. 19 shows, the great majority of the parsers obtain higher LAS than the average LAS over parsers in Zeman et al. [85]. Stanza, Towerparse, and Biaffine consistently outperform the best systems across different languages. Thus, we confirm the legitimacy of the used parsers.

A.2 Sensitivity of metrics to data noise

While our results in §4 indicate that the parsers perform decently on the target corpora, it remains uncertain whether the metrics derived from their parsing results are affected by data noise and to what extent. Since we will inspect the trends of the metrics over time, it is important to ensure that the data noise does not disrupt the rankings of those metrics. Hence, we compute Spearman's ρ correlations between our 15 metrics on the parses of the original and human corrected sentences (without spelling and OCR errors, etc.; collected in Section 3); higher correlations indicate the rankings are less disrupted and vice versa. The results are displayed in Fig. 7.

Metrics	TowerParse	CoreNLP	Stanza	Biaffine	StackPointer	CRF2O	avg
mDD	0.972	0.957	0.983	0.986	0.952	0.971	0.970
nDD	0.987	0.912	0.993	0.983	0.928	0.989	0.966
$Height$	0.993	0.947	0.995	0.992	0.988	0.996	0.985
$Ratio_{head\text{-}final}$	0.988	0.953	0.962	0.988	0.985	0.974	0.975
$treeDegree$	0.985	0.962	0.989	0.991	0.975	0.980	0.980
$\#Leaves$	1.000	0.999	0.999	0.999	0.999	0.999	**0.999**
$degreeVar$	0.993	0.970	0.989	0.994	0.987	0.987	0.986
$degreeMean$	1.000	0.989	0.999	0.999	0.999	0.999	0.998
$depthVar$	0.991	0.940	0.993	0.988	0.985	0.992	0.981
$depthMean$	0.995	0.952	0.994	0.997	0.991	0.998	0.988
$d_{head\text{-}final}$	0.993	0.991	0.990	0.990	0.992	0.992	0.991
$\#Crossings$	0.957		0.964	0.930	0.909	-0.007	<u>0.751</u>
$Height_{dependency}$	0.998	0.958	0.998	0.998	0.997	0.997	0.991
d_{root}	0.999	0.906	0.979	0.974	0.934	0.999	0.965
$d_{randomTree}$	0.992	0.993	0.994	0.990	0.993	0.991	0.992
avg	**0.989**	0.959	0.988	0.987	0.974	<u>0.924</u>	0.970

Table 7. Spearman's ρ between the metrics on the original sentences and that on the human-corrected sentences (collected in Section 3). The "avg" values in the rows refer to the average correlation over different parsers for a specific metric; those in the columns denote the average correlation over different metrics for a specific parser. We bold the highest and underline the lowest average correlations.

A.3 Parser agreements based on mixed-effects regression

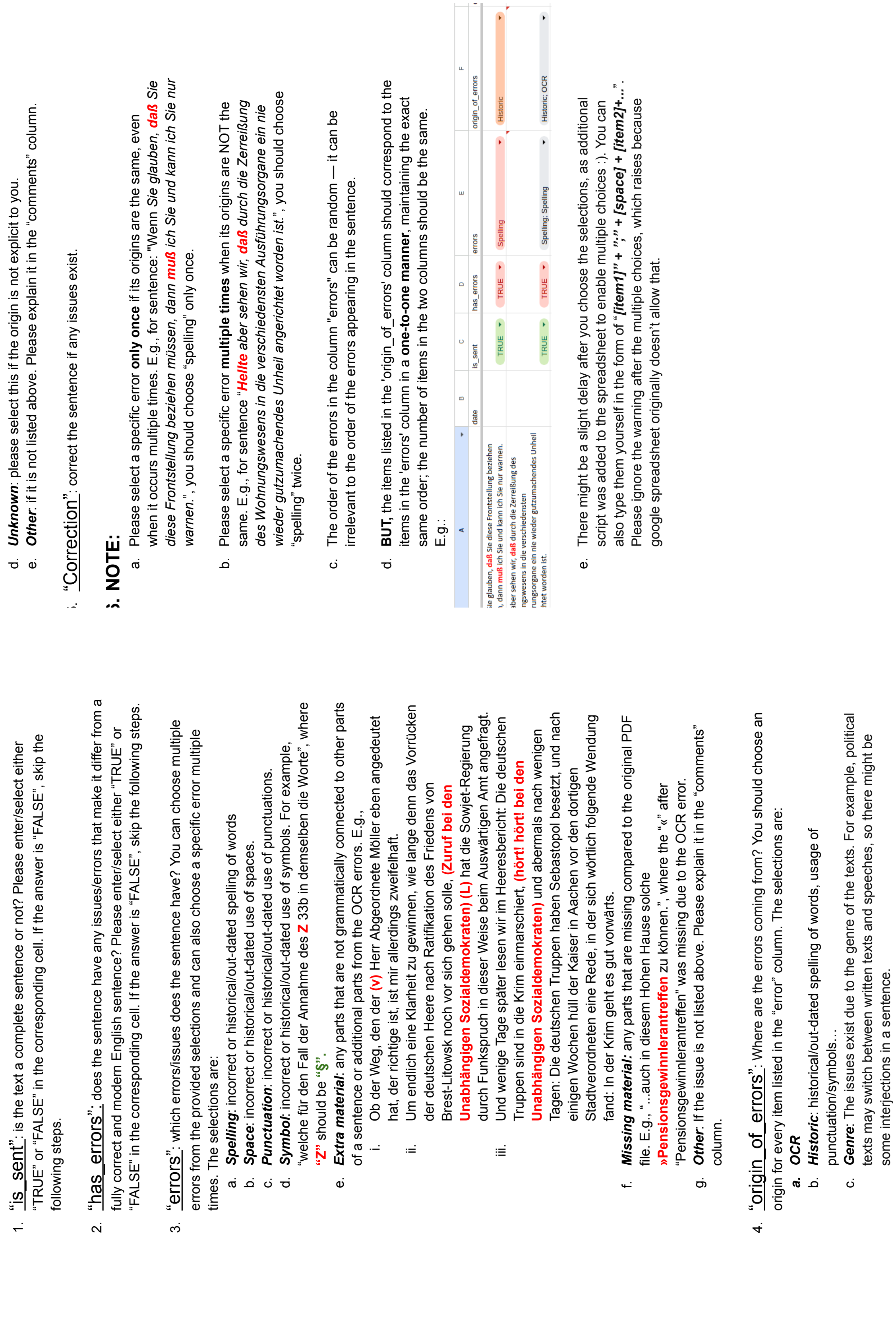

1. “is_sent”: is the text a complete sentence or not? Please enter/select either “TRUE” or “FALSE” in the corresponding cell. If the answer is “FALSE”, skip the following steps.

2. “has_errors”: does the sentence have any issues/errors that make it differ from a fully correct and modern English sentence? Please enter/select either “TRUE” or “FALSE” in the corresponding cell. If the answer is “FALSE”, skip the following steps.

3. “errors”: which errors/issues does the sentence have? You can choose multiple errors from the provided selections and can also choose a specific error multiple times. The selections are:
    a. ***Spelling***: incorrect or historical/out-dated spelling of words
    b. ***Space***: incorrect or historical/out-dated use of spaces.
    c. ***Punctuation***: incorrect or historical/out-dated use of punctuations.
    d. ***Symbol***: incorrect or historical/out-dated use of symbols. For example, “welche für den Fall der Annahme des **Z** 33b in demselben die Worte”, where **“Z”** should be **“§”**.
    e. ***Extra material***: any parts that are not grammatically connected to other parts of a sentence or additional parts from the OCR errors. E.g.,
        i. Ob der Weg, den der **(v)** Herr Abgeordnete Möller eben angedeutet hat, der richtige ist, ist mir allerdings zweifelhaft.
        ii. Um endlich eine Klarheit zu gewinnen, wie lange denn das Vorrücken der deutschen Heere nach Ratifikation des Friedens von Brest-Litowsk noch vor sich gehen solle, **(Zuruf bei den Unabhängigen Sozialdemokraten) (L)** hat die Sowjet-Regierung durch Funkspruch in dieser Weise beim Auswärtigen Amt angefragt.
        iii. Und wenige Tage später lesen wir im Heeresbericht: Die deutschen Truppen sind in die Krim einmarschiert, **(hört! hört! bei den Unabhängigen Sozialdemokraten)** und abermals nach wenigen Tagen: Die deutschen Truppen haben Sebastopol besetzt, und nach einigen Wochen hüll der Kaiser in Aachen vor den dortigen Stadtverordneten eine Rede, in der sich wörtlich folgende Wendung fand: In der Krim geht es gut vorwärts.
    f. ***Missing material:*** any parts that are missing compared to the original PDF file. E.g., “...auch in diesem Hohen Hause solche **»Pensionsgewinnlerantreffen** zu können.”, where the “«” after “Pensionsgewinnlerantreffen” was missing due to the OCR error.
    g. ***Other***: If the issue is not listed above. Please explain it in the “comments” column.

4. “origin_of_errors”: Where are the errors coming from? You should choose an origin for every item listed in the “error” column. The selections are:
    a. ***OCR***
    b. ***Historic***: historical/out-dated spelling of words, usage of punctuation/symbols…
    c. ***Genre***: The issues exist due to the genre of the texts. For example, political texts may switch between written texts and speeches, so there might be some interjections in a sentence.
    d. ***Unknown***: please select this if the origin is not explicit to you.
    e. ***Other***: if it is not listed above. Please explain it in the “comments” column.

[illegible]. “Correction”: correct the sentence if any issues exist.

[illegible]. NOTE:
    a. Please select a specific error **only once** if its origins are the same, even when it occurs multiple times. E.g., for sentence: "Wenn *Sie glauben,* ***daß*** *Sie diese Frontstellung beziehen müssen, dann* ***muß*** *ich Sie und kann ich Sie nur warnen.*”, you should choose “spelling” only once.
    b. Please select a specific error **multiple times** when its origins are NOT the same. E.g., for sentence “***Hellte*** *aber sehen wir,* ***daß*** *durch die Zerreißung des Wohnungswesens in die verschiedensten Ausführungsorgane ein nie wieder gutzumachendes Unheil angerichtet worden ist.*”, you should choose “spelling” twice.
    c. The order of the errors in the column "errors" can be random — it can be irrelevant to the order of the errors appearing in the sentence.
    d. **BUT,** the items listed in the 'origin_of_errors' column should correspond to the items in the 'errors' column in a **one-to-one manner**, maintaining the exact same order; the number of items in the two columns should be the same. E.g.:

A	B	C	D	E	F
	date	is_sent	has_errors	errors	origin_of_errors
ie glauben, daß Sie diese Frontstellung beziehen<br>, dann muß ich Sie und kann ich Sie nur warnen.		TRUE	TRUE	Spelling	Historic
ber sehen wir, daß durch die Zerreißung des<br>ngswesens in die verschiedensten<br>rungsorgane ein nie wieder gutzumachendes Unheil<br>htet worden ist.		TRUE	TRUE	Spelling; Spelling	Historic; OCR

    e. There might be a slight delay after you choose the selections, as additional script was added to the spreadsheet to enable multiple choices :). You can also type them yourself in the form of “***[item1]” + ”;” + [space] + [item2]+...***”. Please ignore the warning after the multiple choices, which raises because google spreadsheet originally doesn’t allow that.

Fig 17. Annotation Guidelines for Hansard.

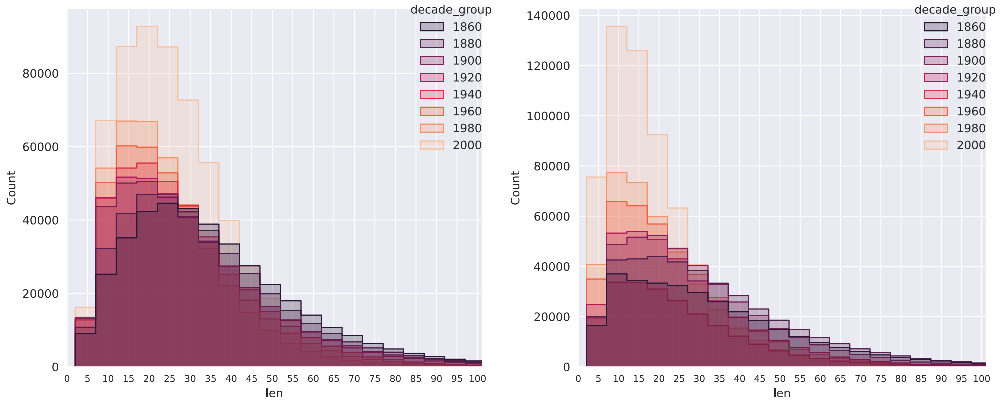

(a) Hansard **(b)** DeuParl

Fig 18. Histogram of sentence length distribution with bin size of 5 for the randomly sampled sentences having at most 100 tokens (which compose 99% of the data). Darker colors indicate older periods and vice versa.

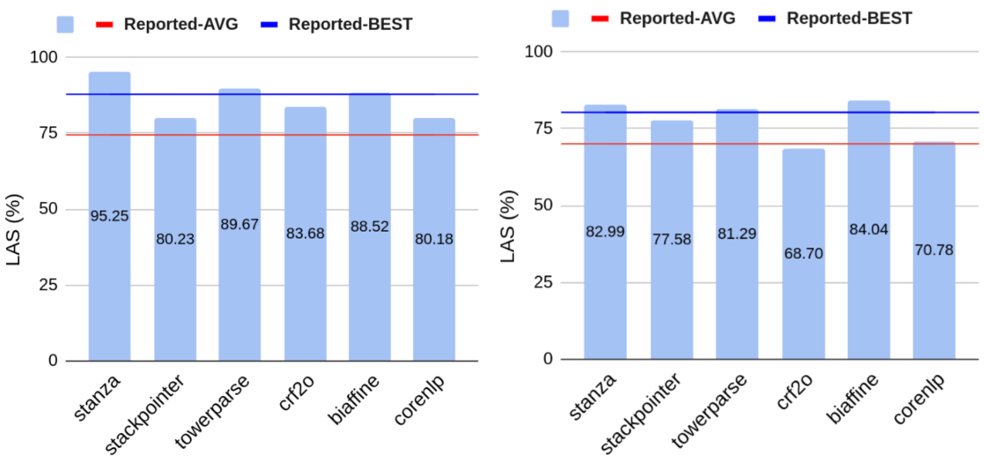

(a) EN-PUD **(b)** DE-GSD

Fig 19. Barplot of LAS of the parsers in our evaluation in comparison to the average and the best LAS over various parsers on the English PUD and German GSD test sets reported in Table 15 of Zeman et al. [85].

Metric	5-7	10-12	15-17	20-22	30-32	40-42	50-52	60-62	70-72
d_{root}	+4	-	-	-	-	-	-	-	-
mDD	-	-	-	-	-	-	-	-	-5
nDD	+6	-	-	-	+6	-	-	-	-
$\#Crossings$	-	-	-	-	-	-	-	-	-
$\#Leaves$	+6	-	-	-	-	-	-	-	-
$Height$	-6	-	-	-	-	+4	-	+6	+5
$Height_{dependency}$	-	-	-	-	-	-	-	-	-
$depthVar$	-6	-	-	+4	-	+4	-	+6	+5
$depthMean$	-6	-4	-	-	-	-	+4	+6	+5
$treeDegree$	+6	-	-	-	-6	-4	-6	-6	-5
$degreeVar$	+6	+4	+6	-	-	-	-5	-6	-5
$depthMean$	+6	-	-	-	-	-	-	-	+6
$Ratio_{head\text{-}final}$	+6	+4	-	-	-	-	-5	-5	-6
$d_{head\text{-}final}$	-6	-	-4	-	-	-	-	-	+4
$d_{randomTree}$	-	-	-	-	-	-	-	-	-

(a) English

Metric	5-7	10-12	15-17	20-22	30-32	40-42	50-52	60-62	70-72
d_{root}	-	-	-	-	-4	-	-	-	-
mDD	+6	+6	+6	+6	-	+6	+5	+5	-
nDD	-6	-6	-	-	-5	-	-	-	-
$\#Crossings$	-	-	-3	-4	-	-	-	-	-
$\#Leaves$	+6	+6	-	-	-5	-6	-6	-6	-6
$Height$	-6	-	-	-	+5	-	+4	+4	+4
$Height_{dependency}$	+6	+6	+4	-	+5	+6	+6	+4	+4
$depthVar$	-6	-	-	-	+4	-	+4	+4	+4
$depthMean$	-6	-4	-	-	+5	+6	+5	+4	+5
$treeDegree$	+6	+6	-	-4	-5	-6	-6	-6	-6
$degreeVar$	+6	+6	-	-4	-5	-6	-6	-6	-6
$degreeMean$	+4	+6	-	-	-	-4	-4	-5	-5
$Ratio_{head\text{-}final}$	-	-	-	-	-5	-6	-6	-6	-6
$d_{head\text{-}final}$	-6	-5	-	-	+5	+6	+6	+6	+6
$d_{randomTree}$	-	-	-	-	-	-	-	-	-

(b) German

Table 8. Metrics that have a stable trend supported by at least 3 parsers (except for $\#Crossings$, for which we relax the minimal requirement to 2 parsers, as CoreNLP is unable to predict crossing edges.). "+" and "−" denote the increasing and decreasing trend respectively; the values refer to the number of parsers that support this trend.

parser1	parser2	en	de
biaffine	corenlp	0.475	0.498
biaffine	crf2o	0.575	0.699
biaffine	stackpointer	0.576	0.699
biaffine	stanza	0.712	0.677
biaffine	towerparse	0.490	0.733
corenlp	crf2o	0.436	0.482
corenlp	stackpointer	0.486	0.525
corenlp	stanza	0.533	0.572
corenlp	towerparse	0.272	0.409
crf2o	stackpointer	0.481	0.795
crf2o	stanza	0.571	0.704
crf2o	towerparse	0.515	0.605
stackpointer	stanza	0.538	0.749
stackpointer	towerparse	0.256	0.695
stanza	towerparse	0.463	0.672
avg		0.492	0.634

Table 9. Cohen's Kappa of mixed-effects regression based on the dependency relations predicted by different parsers.
